# Recent Advances for the Synthesis and Applications of 2-Dimensional Ternary Layered Materials

**DOI:** 10.34133/research.0040

**Published:** 2023-01-30

**Authors:** Jing Peng, Zheng-jie Chen, Baofu Ding, Hui-Ming Cheng

**Affiliations:** ^1^ Faculty of Materials Science and Energy Engineering/Institute of Technology for Carbon Neutrality, Shenzhen Institute of Advanced Technology, Chinese Academy of Sciences, Shenzhen 518055, China.; ^2^ Shenzhen Key Laboratory of Energy Materials for Carbon Neutrality, Shenzhen Institute of Advanced Technology, Chinese Academy of Sciences, Shenzhen 518055, China.; ^3^ Shenyang National Laboratory for Materials Science, Institute of Metal Research, Chinese Academy of Sciences, Shenyang 110016, China.

## Abstract

Layered materials with unique structures and symmetries have attracted tremendous interest for constructing 2-dimensional (2D) structures. The weak interlayer interaction renders them to be readily isolated into various ultrathin nanosheets with exotic properties and diverse applications. In order to enrich the library of 2D materials, extensive progress has been made in the field of ternary layered materials. Consequently, many brand-new materials are derived, which greatly extend the members of 2D realm. In this review, we emphasize the recent progress made in synthesis and exploration of ternary layered materials. We first classify them in terms of stoichiometric ratio and summarize their difference in interlayer interaction, which is of great importance to produce corresponding 2D materials. The compositional and structural characteristics of resultant 2D ternary materials are then discussed so as to realize desired structures and properties. As a new family of 2D materials, we overview the layer-dependent properties and related applications in the fields of electronics, optoelectronics, and energy storage and conversion. The review finally provides a perspective for this rapidly developing field.

## Introduction

The development of modern society is inseparable from 3 pillars—energy, information, and material. As the carrier of energy and information, new material is an indispensable driving force for the development of modern society. After entering the 21st century, the design and exploration of new functional materials that break the traditional framework have been put into a particularly prominent position so as to satisfy people's urgent demands for technological breakthrough and sustainable development. Since Andre Geim et al. isolated graphite layers into graphene in 2004, such layered solids formed by van der Waals (vdW) forces between interlayers have attracted extensive attention to create a new realm of materials [1–5]. Two-dimensional (2D) materials possess unique atomic thickness and infinite planar structure, which enable particles or quasiparticles such as electrons, excitons, and magnons to exhibit exotic behaviors differing from their 3D bulk counterparts stemming from the quantum confinement effect [6–9]. These novel and unique properties in 2D structure have opened the "2-dimensional era", and brought great potential in various applications including electronics, optoelectronics, and energy conversion and storage.

Many 2D materials have been explored and investigated, including graphene, h-BN, and transition metal dichalcogenides (TMD), and various top-down and bottom-up ways are proposed and applied to synthesize them [10–13]. While these elemental and binary materials suffer from fewer types and combinations for chemical composition, only a few dozen 2D materials have been successfully synthesized, and expanding the library of 2D materials becomes necessary and urgent. In recent years, ternary layered materials have attracted tremendous interests, since they greatly enrich the members of the 2D realm [14–18]. When compared to elemental and binary materials the addition of an element introduces new compositional combinations and layer arrangement styles, which bring about a variety of novel materials with accompanied physical and chemical properties. For example, by introducing Mn into a binary topological insulator, Bi_2_Te_3_, the ternary layered MnBi_2_Te_4_ can be achieved with intrinsic magnetic topological insulation and realize a quantum anomalous Hall effect [19]. The ternary materials can be extended into a series of vdW compounds via changing the amount of Mn, such as MnBi_4_Te_7_ and MnBi_6_Te_10_ [20]. In addition, as a ternary layered material is thinned to a 2D structure, its chemical composition and lattice structure can be modulated and even used to create new materials with altered stoichiometric ratio or crystal symmetry [21,22]. For layered transition metal carbides/nitrides (M_*n*+1_AX*_n_*), they can be exfoliated into ultrathin nanosheets with a chemical formula of M_*n*+1_X*_n_*T*_x_*, where T is surface functional groups [23]. Furthermore, 2D ternary layered materials can be constructed by means of covalent coupling of 2 binary compounds, where multifunctional materials can be designed with properties inheriting from their parents [15,24]. Taking VSi_2_N_4_ as an example, it can be viewed as the coupling of VN_2_ and SiN layers and possesses unique semiconducting ferromagnetism [25]. Therefore, the exploration of ternary layered materials can not only offer opportunities in extensively exploring new 2D materials with abundant and exotic properties but also provide new paradigms to design and construct functional materials with desired electronic structures.

**Fig. 1.**
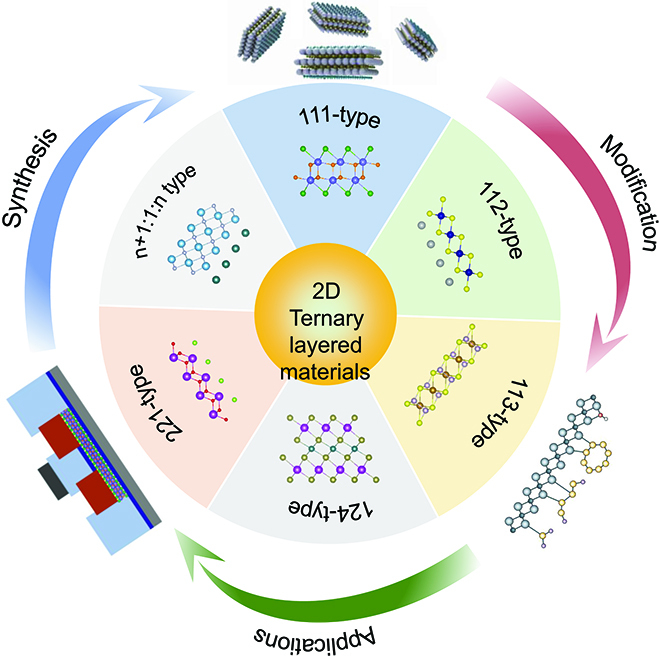
Schematic of the synthesis, modification, and applications of 2D ternary layered materials with different stoichiometric ratios and structures.

To date, there have been a lot of bulk ternary layered materials discovered with different chemical compositions and stoichiometric ratios, such as ternary layered oxides, transition metal phosphorus trichalcogenides, and MAX phase materials. These materials possess various electronic properties and contain metals, semiconductors, magnets, and topologic insulators. Thus, the derived 2D materials emerge diverse physical and chemical properties, which show great potential for applications in many fields. Differing from elemental and binary layered materials, more elements in the periodic table are involved in the formation of ternary compounds, and the complicated compositions and structures give rise to more flexible regulation; thereby, more possibilities and challenges exist toward the preparation and investigation of 2D ternary layered materials. We conduct a comprehensive review of ternary layered compounds and corresponding 2D materials. We first classify them by virtue of the stoichiometric ratio and then emphasize the recent progress made in the synthesis and modification of 2D ternary materials. Furthermore, the synthesis approaches are discussed for ternary layered materials with different interlayer interactions. As a new catalog of 2D materials, the intrinsic properties and potential applications have been summarized (Fig. 1). The review finally provides an outlook of the challenges and future for this rapidly developing field.

## Classifications

**Fig. 2.**
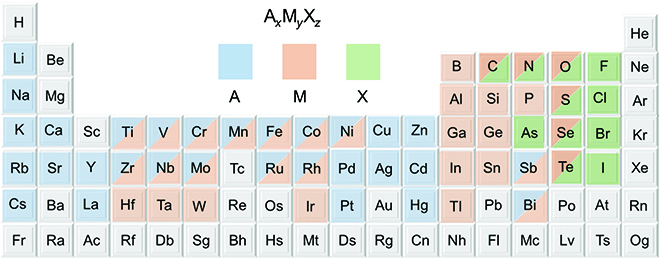
Hundreds of different ternary layered compounds exist. The metals and non-metal elements that predominantly crystallize in layered structures with different stoichiometric ratios are highlighted in the periodic table. Some elements can occupy 2 kinds of positions in A*_x_*M*_y_*X*_z_*.

To date, large quantities of ternary layered materials have been explored, with their lattices and electronic structures being investigated. They exhibit several structural characteristics: (a) a layered structure with strong anisotropy; (b) strong chemical bonds connecting in intralayers, while relatively weak bonds and even the vdW interaction between interlayers; (c) at least triple atomic layers to form a monolayer; and (d) the atomic layers of 3 elements are arranged individually or in pairs and interpenetrated between interlayers. Since the ternary layered materials can be made up of nearly all the non-radioactive elements with multiple valence states, they form abundant compounds with different chemical compositions and stoichiometric ratios (Fig. 2). In this section, we classify these ternary layered materials in terms of stoichiometric ratios.

### 111-Type compounds

**Fig. 3.**
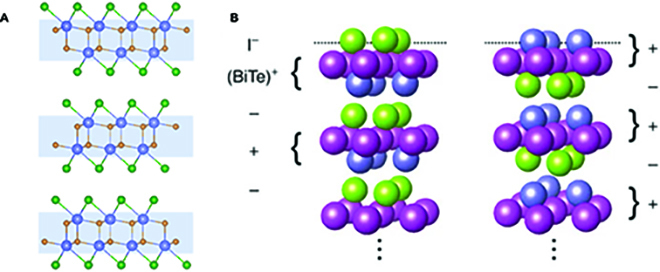
(A) Crystal structure of ZrNCl. Most 111-type compounds show a slightly different structure between 2 halogen layers, which highlights in the blue block. (B) Two expected surface termination structures of BiTeI with labeled charges in the ionic limit. Part (B) is reproduced with permission from Ref. [31], Copyright 2014, Nature Publishing Group.

There is a large class of layered materials exhibiting a stoichiometric ratio of 1:1:1. In general, these materials consist of one metallic element and 2 nonmetallic elements, one of which is halogen. The 111-type compounds mainly include metal oxyhalides (such as BiOCl, VOCl, and CrSBr) [26,27], metal nitride halides (such as ZrNCl and HfNBr, Fig. 3A), and metal carbide halides (such as La_2_C_2_Br_2_ and Y_2_C_2_I_2_) [28,29]. They are composed of a layered structure, where each monolayer contains a fluorite-type [M_2_O_2_], [M_2_N_2_], or [M_2_C_2_] layer and 2 layers of halogen forming a typical sandwich structure. However, with respect to BiTeX compounds (Fig. 3B), the Bi, Te, and X layers are stacked alternately with the monolayer structure of Bi-Te-X, so that the mirror and inversion symmetries along the *c* axis are broken, which triggers a giant Rashba effect in the electronic structure of such semiconducting layered materials [30,31]. Given that each layer of 111-type compounds is stacked together by the vdW force, they can be mechanically exfoliated into atomic-thick 2D nanosheets.

Owing to different chemical compositions, 111-type compounds exhibit diverse electronic and optoelectronic properties. Most metal oxyhalides and nitride halides are semiconductors, while metal carbide halides exhibit metallic and even superconducting behavior. For instance, BiOCl is a semiconductor with a bandgap of 3.2 eV, which can serve as an efficient photocatalyst to decompose organic pollutants and nitrogen reduction reaction [32,33], and the layered Y_2_C_2_I_2_ possesses superconductivity at a critical temperature of 9.97 K [34]. Moreover, some compounds containing magnetic elements (V, Cr, and Fe) show intrinsic ferromagnetism or antiferromagnetism, thereby providing candidates for robust 2D magnetic materials [27,35].

### 112-Type compounds

Ternary layered materials with a stoichiometric ratio of 1:1:2 are usually endowed with the general chemical formula of AMX_2_, where A is monovalent ion including alkali metal and IB metals, and M is trivalent metal ions and the anion is chalcogen or nitrogen. These compounds generally have the *R3m* space group, and the structure consists of a layer of A sandwiched between 2 layers of MX_2_. Hence, they can be regarded as intercalation compounds of layered metal dichalcogenides formed by A ion insertion. According to the composition of A and M, A ions can occupy the interstices constructed by neighboring anion layers with 3 formats, which are tetrahedral sites, octahedral sites, and linear dumbbells.

**Fig. 4.**
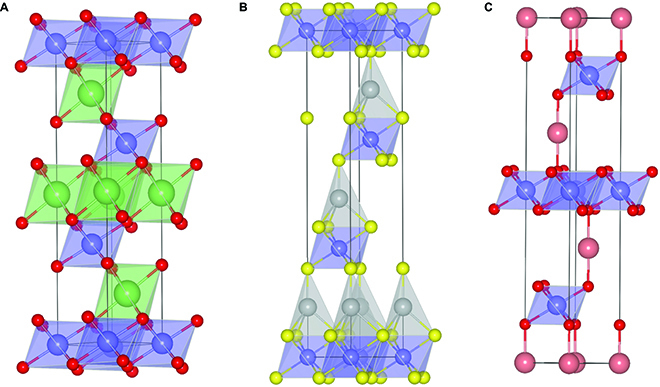
Crystal structures of LiCoO_2_ (A), AgCrS_2_ (B), and PdCoO_2_ (C). The green, gray, and pink balls in (A), (B), and (C) are Li, Ag, and Pd, respectively. The linked type of A sites are octahedral interstices, tetrahedral interstices, and linear dumbbells for LiCoO_2_, AgCrS_2_, and PdCoO_2_, respectively.

A representative material of AMX_2_ compounds is LiCoO_2_, a commercially prevailing cathode material for lithium-ion batteries [36,37]. The layered structure is composed of closed-packed oxygen planes stacked in an ABC sequence, with Co and Li ions residing in octahedral sites in alternating oxygen layers (Fig. 4A). Other metal dichalcogenides, such as LiVS_2_, AgCrS_2_, NaCrSe_2_, and CuInS_2_, show a similar structure to LiCoO_2_, whereas the species of A ions determine the occupied position between anion layers [38,39]. As the A element belongs to alkali metal, A ions reside in octahedral sites, while half of the tetrahedral sites are occupied when A is IB metal (Fig. 4B). With respect to delafossite-type materials, such as CuFeO_2_ and PdCoO_2_, the A ions are interlinked by linear O–A–O dumbbells parallel to the *c* axis [40] (Fig. 4C). Except for layered oxides or dichalcogenides, nitrides can also form ternary layered materials with a chemical formula of AMN_2_, where A can be a multivalent metal and M is an IVB–VIB transition metal [41]. The ternary transition metal nitrides such as FeWN_2_, LiMoN_2_, and SrHfN_2_ are hexagonal layered materials similar in structure to LiCoO_2_ with an alternating arrangement of A atoms and N–M–N layers that coordinates to 6 nitrogen atoms in a trigonal prismatic coordination.

Owing to the chemical bonds with strong interaction between interlayers of AMX_2_ compounds, great challenge exists in the synthesis and exploration of their 2D materials. Nevertheless, many 112-type layered compounds can serve as suitable host materials to reverse insertion and de-intercalation for A ions. The unique feature is of great importance for energy storage, and is also utilized to synthesize 2D binary oxides or chalcogenides [36,42]. Since the alkaline metal ions residing in AMX_2_ layers show high activity with water, gigantic swelling between MX_2_ layers will occur accompanied by hydrogen generation and water molecule intercalation, which results in efficient exfoliation of AMX_2_ and achieves MX_2_ nanosheets [43,44].

### 113-Type compounds

**Fig. 5.**
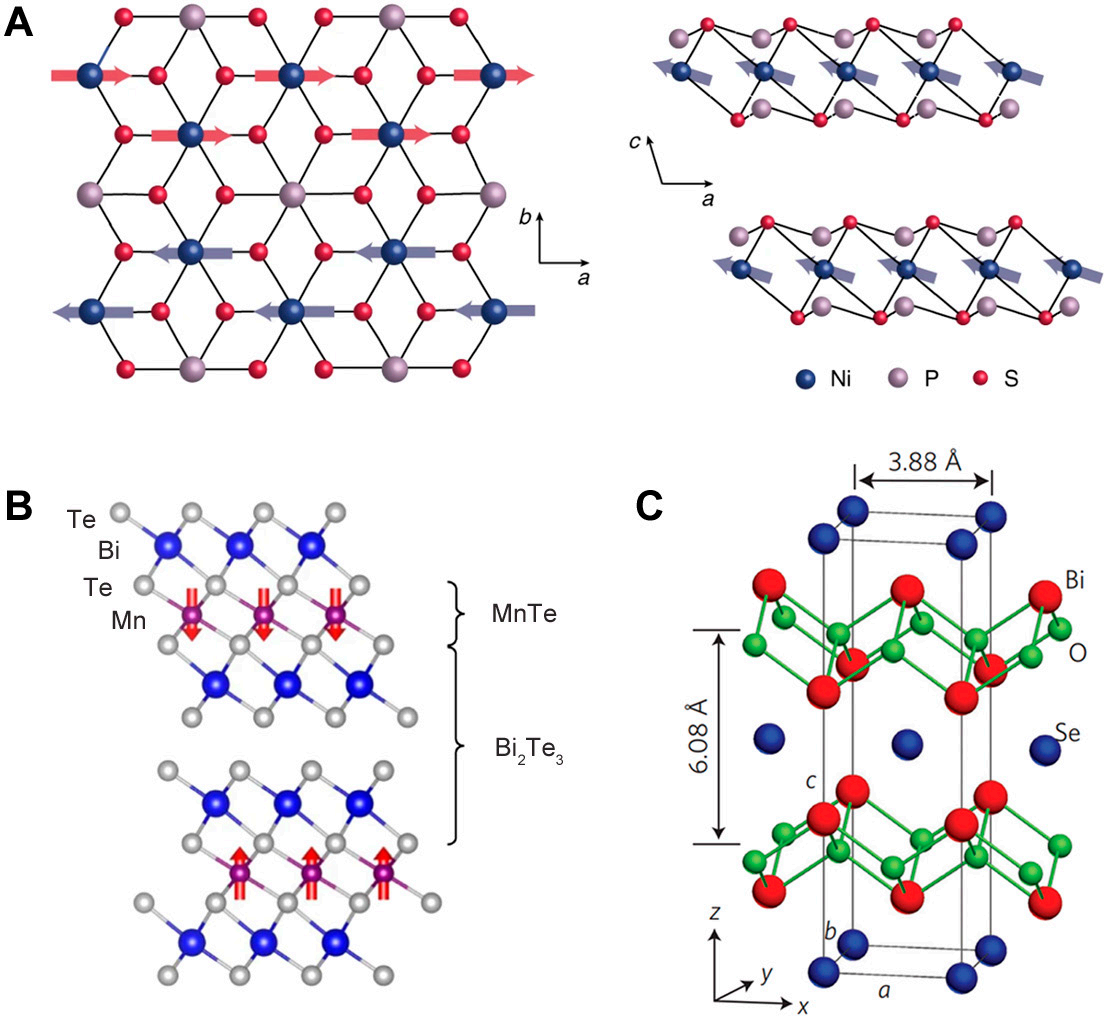
(A) Crystal and magnetic structures of NiPS_3_ along the *ab* plane and *c* direction. (B) Crystal structures of MnBi_2_Te_4_. (C) Crystal structure of Bi_2_O_2_Se with tetragonal (Bi_2_O_2_)^2+^*_n_* layers and Se^2−^*_n_* layers alternately stacked along the *c* axis. Panel (A) is reproduced with permission from Ref. [47], Copyright 2021, Nature Publishing Group. Panel (B) is reproduced with permission from Ref. [50], Copyright 2020, Nature Publishing Group. Panel (C) is reproduced with permission from Ref. [17], Copyright 2017, Nature Publishing Group.

113-Type ternary layered materials mainly contain transition metal phosphorus trichalcogenides (MPX_3_, X = S and Se), such as FePS_3_, MnPSe_3_, and NiPS_3_ (Fig. 5A) [45]. In the MPX_3_ crystal, the CdCl_2_-type structure is built, in which honeycomb configured metal ions are disseminated around the [P_2_X_6_] bipyramids [46,47]. Therefore, these materials can be viewed as a salt composed of M^2+^ cations and [P_2_X_6_]^4−^, endowed with the type of M_2_P_2_X_6_. Besides MPX_3_, layered CrSiTe_3_ and Cr_2_Ge_2_Te_6_ also have a 1:1:3 stoichiometric ratio [48,49]. The interlayer interaction of 113-type materials is vdW force, leading to their ready exfoliation into atomic-thick nanosheets. Most of their bulk are semiconductor or insulator, and intrinsic magnetism can be found when the metal ion is a magnetic element. After exfoliation, robust ferromagnetism or anti-ferromagnetism is uncovered in Cr_2_Ge_2_Te_6_ or FePS_3_, which shows great potential in novel spintronic devices and magnetic memory storage [16,49].

### 124-Type compounds

124-Type layered materials are vdW materials. One of the typical 124-type compounds is MnBi_2_Te_4_-related ternary chalcogenides, which crystallize in the tetradymite-type structure with the *R3m* space group. Taking MnBi_2_Te_4_ as an example, each monolayer of MnBi_2_Te_4_ consists of a septuple-layer block along the *c* axis, with the sequence of Te-Bi-Te-Mn-Te-Bi-Te [15,50] (Fig. 5B). It can also be viewed as a Mn-Te layer inserted into the middle of 2 Bi_2_Te_3_ layers. Since Bi_2_Te_3_ is a topological insulator and Mn^2+^ is in high-spin configuration with 5 *μ_B_* magnetic moment, MnBi_2_Te_4_ is testified to be a topological insulator with intrinsic magnetic order, which shows a quantum anomalous Hall effect when it is mechanically exfoliated into atomically thin layers [19].

Recently, a new class of 124-type layered materials, the MoSi_2_N_4_ family, has been discovered and shows diverse electronic and optoelectronic properties [14,24,51]. They are also built up by septuple atomic layers, which consist of a MoN_2_ layer sandwiched between 2 Si-N bilayers. The structure is similar to that of MnBi_2_Te_4_, but there have been no experimentally known 3D parent crystals before the 2D MoSi_2_N_4_ family was synthesized by using chemical vapor deposition (CVD). Therefore, the discovery of the MoSi_2_N_4_ family has opened up a new paradigm to synthesize 2D materials that have no known 3D layered allotropes. Theoretical calculations showed that the MoSi_2_N_4_ family has a broad class of 2D vdW layered materials with a general formula of MA_2_Z_4_, where M can be transition metal elements of groups IV to VIB, A is an element of group IVA, and Z is an element of groups V to VIA. These materials have been predicted to exhibit unusual electronic structures, and thus, topological, magnetic, and superconducting properties are promising to emerge, which require further experimental exploration.

### 221-Type compounds

The bismuth oxyselenides (Bi_2_O_2_X, X is chalcogen) are a group of ternary layered materials with a stoichiometric ratio of 2:2:1. They can be considered to partially replace stoichiometric X atoms of layered Bi_2_X_3_ with lighter O atoms. The Bi_2_O_2_X is a layered material with a tetragonal crystal structure, which consists of strongly bonded [Bi_2_O_2_]^2+^ and Se^2−^ square net layers alternately stacked along the *c* axis with electrostatic interactions between them (Fig. 5C). Despite the relatively weak interlayer interaction compared to in-plane covalent bonds, it is hard to achieve large and ultrathin nanosheets through direct exfoliation of such non-vdW layered materials. As an emerging material, Bi_2_O_2_X shows excellent electronic properties with a high carrier mobility of over 20,000 cm^2^ V^−1^ s^−1^, thereby making it promising for high-performance electronics and optoelectronics [17,52 ,53].

### (*n*+1):1:*n* compounds (*n* = 1, 2, 3)

**Fig. 6.**
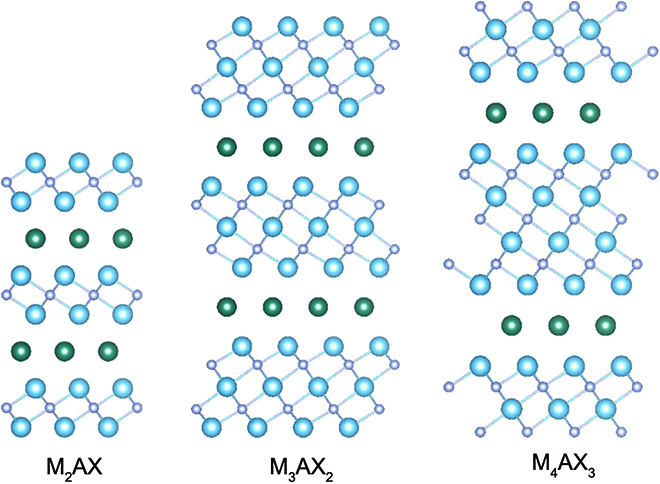
Crystal structures of MAX phase materials, where the blue, green, and purple balls represent M, A, and X atoms, respectively.

There is a big family of ternary carbide and nitride compounds exhibiting a chemical formula of “M_*n*+1_AX*_n_*” with the same layered structure, where M stands for an early transition metal, A is an A-group element, X corresponds to carbon or nitrogen, and *n* is equal to 1 to 3 (Fig. 6). Owing to the general formula, they are simplified to “MAX” and lead to the known MAX phase materials [54]. All of the MAX phases present a hexagonal crystal structure with a space group of *P63*/*mmc*, and the layers of edge-shared M_6_X-octahedra are alternately stacked with A layers along the *c* axis. Although the M–A bonds are relatively weaker than that of the M–X bonds with the metallic-covalent nature, the layered MAX phase cannot be directly exfoliated into 2D nanosheets.

As early as the 1960s, the MAX phase materials had gained great attention with their unique physical and mechanical properties including excellent electrical conductivity, good thermal conductivity, machinability, and high oxidation and corrosion resistance, which can be used in the fields of aerospace, manufacturing industry, nuclear industry, and so on [55]. Recently, a new class of 2D materials based on the MAX phase has been exploited through selective etching [56]. Owing to the removal of A-group atoms, M_*n*+1_X*_n_* nanosheets are achieved and denoted as MXene. Compared to the bulk counterparts, MXenes combine the metallic conductivity and stability of MAX phase and are simultaneously endowed with a much larger surface area stemming from atomic thickness. Thus, this new type of materials exhibits promising performance in energy storage and conversion [18].

Besides the above 6 kinds of ternary layered materials with well-defined stoichiometry, there are a series of non-stoichiometric layered alloys consisting of 3 elements. It is widely known that alloying is an efficient tool to finely engineer the lattice and electronic structures of materials, especially the semiconducting metal chalcogenides [57,58]. During the alloying process, lattice strain will be introduced because of the different lattice parameter for the corresponding binary compounds. In order to reduce lattice mismatch, alloying within the same metal or nonmetallic groups is favorable. Currently, transition metal chalcogenide alloys including Mo*_x_*W_(1−*x*)_S_2_, Zr*_x_*Hf_(1−*x*)_S_2_, MoS_2(1−*x*)_Se_2*x*_, and Ga*_x_*In_(1−*x*)_Se have been successfully synthesized [59–61]. They exhibit layered features with the same lattice symmetry and coordination structure with parent counterparts. Generally, the substituted atoms are randomly distributed in the intralayers due to the large entropy, and thus, both bulk and their 2D materials form the random phases. By gradually changing the compositional components, the electronic and optoelectronic properties can be continuously modulated. For example, the photoluminescence emission wavelengths of MoS_2(1−*x*)_Se_2*x*_ monolayer alloys can be continuously tuned and nearly linearly vary from 670 to 800 nm as the Se content increases [58]. Besides randomized alloys’ structure, alloyed 2D materials with ordered atomic substitution can be grown and exhibit unique structure and property. Lu et al. [62] reported a Janus MoSSe monolayer with the Mo layers sandwiched by the bottom S and top Se atoms. Owing to the out-of-plane structural symmetry being broken, the Janus MoSSe induces vertical dipoles and out-of-plane piezoelectricity emerges. Therefore, structural control of alloyed 2D materials is vital to their electronic properties.

## Synthesis of 2D Materials

Generally, as layered materials are thinned down from bulk to 2D nanosheets, their electronic structures will change remarkably and distinct properties will emerge due to the quantum confinement effect. Similar to previously reported elemental and binary layered materials, ternary layered materials with strong anisotropy can provide great potential to obtain their 2D structures, so as to investigate the variation of structure and property at the 2D limit. Compared to the former, ternary compounds consist of richer chemical components and stoichiometric ratios, and their lattice structures are more complicated. The sophisticated chemical and structural nature produces more exotic electronic and optical properties, but brings in great challenges in preparation, especially for the 2D form. In addition, the interlayer interaction is distinct in different ternary layered materials. When the layers are constructed by a weak vdW force, the 2D materials can be produced through top-down exfoliation. However, materials with chemical bonds between layers are difficult to isolate directly, and thus, artificially constructing vdW gap through selective etching is a prerequisite. Aside from top-down strategies, bottom-up routes are also used to synthesize 2D materials. However, it is difficult to precisely control the growth of multicomponent materials with orderly atomic arrangements. Furthermore, the terminated groups of non-vdW materials are constructed by rich unsaturated atoms, which makes it harder to control the thickness of 2D sheets during the growth process. Herein, we classify the ternary materials into 2 parts to discuss the preparation of 2D ternary materials: vdW and non-vdW layered materials (Table).

**Table.** A summary of ternary layered materials and related synthesis methods

Stoichiometric ratio	Materials	Interlayer interaction	Synthesis of 2D materials
111	FeOCl, ZrNCl, La_2_C_2_Br_2_, etc.	van der Waals force	• Mechanical exfoliation
			• Solution-based exfoliation
			• Chemical vapor deposition
			• Wet chemical synthesis
112	LiCoO_2_, AgCrS_2_, CuFeO_2_, etc.	Ionic or covalent bonds	• Wet chemical etching and exfoliation
			• Electrochemical etching and exfoliation
113	FePS_3_, Cr_2_Ge_2_Te_6_, etc.	van der Waals force	• Mechanical exfoliation
			• Solution-based exfoliation
124	MnBi_2_Te_4_, MoSi_2_N_4_, etc.	van der Waals force	• Mechanical exfoliation
			• Chemical vapor deposition
			• Molecular beam epitaxy
221	Bi_2_O_2_Se, etc.	Covalent bonds	• Chemical vapor deposition
*n*+1:1:*n*	Ti_2_AlC, Ti_3_AlC_2_, V_4_AlN_3_, etc.	Covalent bonds	• Wet chemical etching and exfoliation
			• Thermal etching and exfoliation
			• Electrochemical etching and exfoliation

### vdW ternary layered materials (111-, 113-, and 124-type)

Many ternary layered materials are held by a weak interlayer interaction, including 111-, 113-, and 124-type compounds. Similar to graphite and transition metal dichalcogenides, the terminated atoms in each monolayer are nonmetallic elements, and the layers are constructed by vdW force. Therefore, they can be easily laminated into a 2D structure via exfoliation, which is utilized to largely increase interlayer distance and weaken the vdW force, thereby producing single- or few-layer nanosheets. To date, various exfoliation approaches have been performed to tear vdW ternary materials, including the well-known mechanical exfoliation, sonication-assisted liquid exfoliation, and chemical exfoliation.

#### Mechanical exfoliation

Mechanical exfoliation is a clean and conventional route to produce single-crystal nanosheets with high quality and low defect density, which is appropriate for the exploration of their intrinsic characteristics. With respect to various vdW ternary materials, their physical properties remain unknown when reduced to atomic thickness and need to be uncovered through mechanical exfoliation. To date, a few 2D ternary materials have been peeled from their parent bulk crystals by using mechanical cleavage, including metal oxide/nitride halides [35,63–65], metal phosphorus trichalcogenides [16,66], and MnBi_2_Te_4_-related ternary chalcogenides [19,67]. Gong et al. [49] adopted mechanical exfoliation to obtain pristine Cr_2_Ge_2_Te_6_ atomic layers and proved intrinsic long-range ferromagnetic order surviving in such 2D vdW crystals, whose transition temperature can be easily controlled using very small fields. Zhang’s group developed an Al_2_O_3_-assisted mechanical exfoliation technique, achieving atomically thin MnBi_2_Te_4_. They found that the MnBi_2_Te_4_ flake becomes ferromagnetic when it has an odd number of septuple layers and observed a zero-field quantum anomalous Hall effect in a 5 septuple-layer sample at 1.4 K [19].

**Fig. 7.**
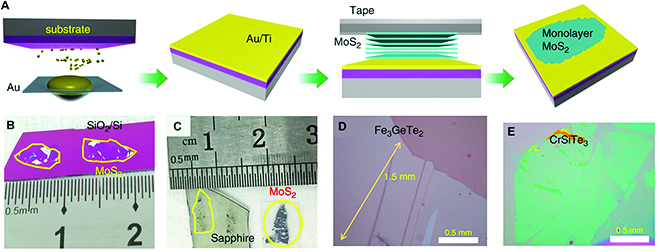
Au-assisted mechanical exfoliation of different monolayer materials with a macroscopic size. (A) Schematic of the exfoliation process. (B) and (C) Optical images of exfoliated MoS_2_ on SiO_2_/Si and sapphire. (D and E) Optical images of large exfoliated 2D ternary layered crystals: Fe_3_GeTe_2_ and CrSiTe_3_. Reproduced with permission from Ref. [68], Copyright 2020, Nature Publishing Group.

It is known that mechanical exfoliation often suffers from small lateral sizes and low yield of the resultant 2D flakes. To resolve these issues, a modified exfoliation method has been proposed through a covalent-like quasi-bonding strategy [68]. A thin layer of Au is deposited on a substrate to realize robust 2D crystal/Au interaction (Fig. 7A), which is sufficient to overcome the interlayer attraction and thus facilitate exfoliating millimeter-sized monolayers from a broad range of layered crystals. From Fig. 7B to E, ternary layered materials including CrSiTe_3_ and Fe_3_GeTe_2_ can be exfoliated into monolayers with quite large lateral sizes.

#### Solution-based exfoliation

Solution-based synthesis of 2D materials is a universal approach for various vdW layered solids with low-cost and mass production, which mainly includes mechanical force-assisted liquid exfoliation and chemical exfoliation. Given the fact that the in-plane structure of layers is made up of chemical bonds with a higher stiffness than that of vdW force between interlayers, external mechanical forces such as sonication and shear forces can be carried out to overcome the interlayer interaction without breaking intralayer bonds, thereby achieving single- and few-layer nanosheets. For the chemical intercalation and exfoliation strategy, vdW layered materials act as a host to allow molecules, ions, or atoms to be inserted into layers, so as to widen the interlayer distance. As a result, the weakened vdW force can be easily broken by sonication or shaking, which leads to the efficient production of monolayers.

**Fig. 8.**
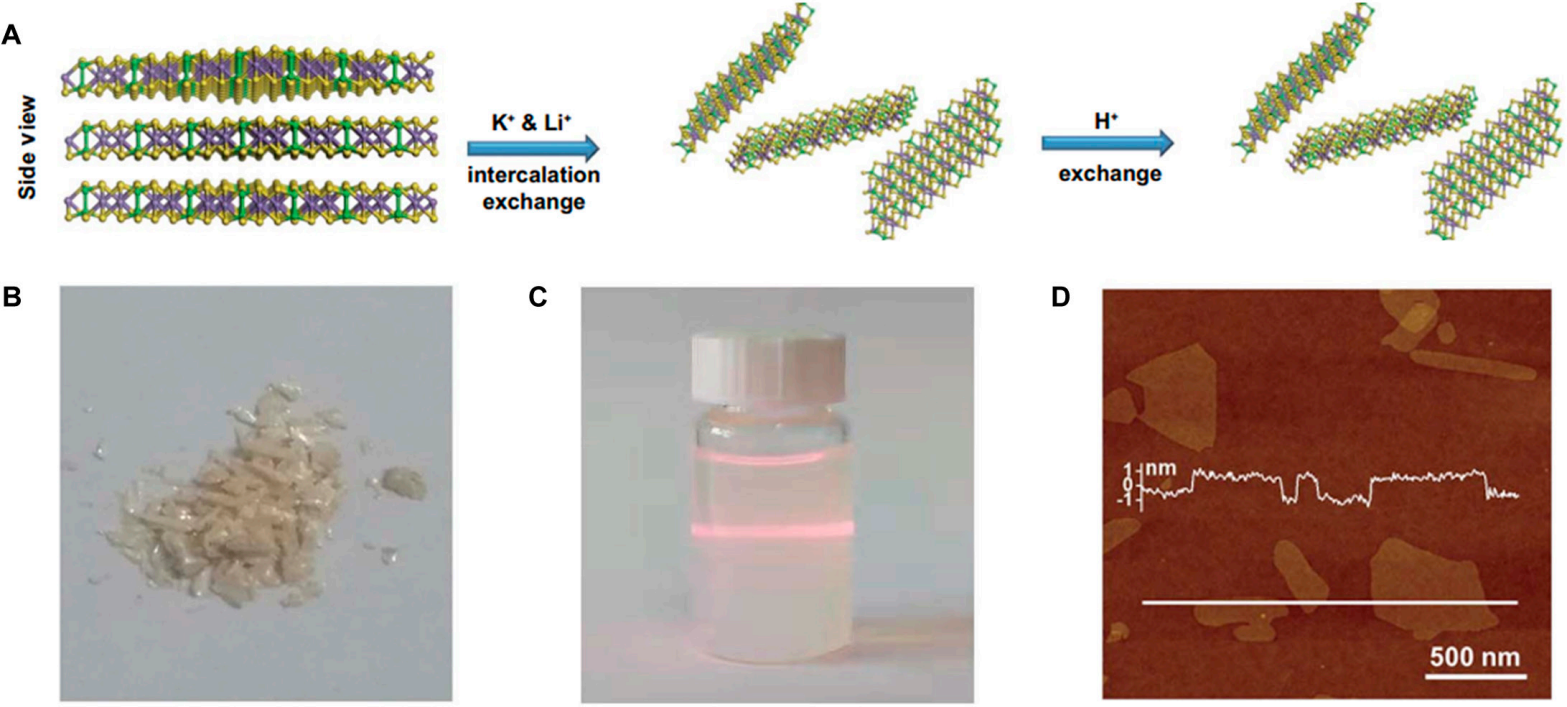
Alkaline ion intercalation exchange to exfoliate and synthesize CdPS_3_ nanosheets. (A) Schematic of the synthesis process. (B) CdPS_3_ crystals. (C) Cd_0.85_PS_3_Li_0.15_H_0.15_ nanosheet water dispersions. (D) A typical atomic force microscope (AFM) image of Cd_0.85_PS_3_Li_0.15_H_0.15_ nanosheets with a thickness of ~1 nm. Reproduced with permission from Ref. [76], Copyright 2020, American Association for the Advancement of Science.

Since the ternary layered materials such as 111-, 124-, and 113-type compounds are constructed by a weak vdW force between neighboring layers, both solution-processing routes can be used to exfoliate them into atomic thin nanosheets [69–76]. For example, 111-type FeOCl is found to possess similar cleavage energies (340 mJ m^−2^) with graphite (320 mJ m^−2^), and a facile ultrasonication-assisted liquid-phase exfoliation route is developed to prepare few-layer FeOCl nanosheets in acetonitrile [70]. In addition, as the most commonly used guest species, lithium ion is developed to chemically intercalate and exfoliate ternary layered materials, including ZrNCl and metal phosphorus trichalcogenides. Prominently, Qian et al. [76] have synthesized CdPS_3_ nanosheets with substantial transition metal vacancies through an alkaline ion intercalation exchange route followed by exfoliation (Fig. 8). The cadmium vacancies in the 2D structures are found to enable the obtained Cd_0.85_PS_3_Li_0.15_H_0.15_ films with exceptionally high proton conductivity. It is worth noting that many ternary layered materials consist of metal elements with multiple oxidation states, such as Fe, Co, and Ni, while they could be reduced into low-valent or even elemental states during the chemical intercalation process because of a large amount of charge transfer. Consequently, compositional alteration and structural reorganization are always accompanied by the as-exfoliated nanosheets. In order to avoid an excessive reduction, nitrogen-containing molecules are adopted to intercalate ternary layered materials with less charge injected. In this case, organic amine and hydrazine are introduced to chemically exfoliate FePS_3_, and ultrathin nanosheets can be efficiently produced with different lateral sizes [74,77].

#### Solid-state growth

**Fig. 9.**
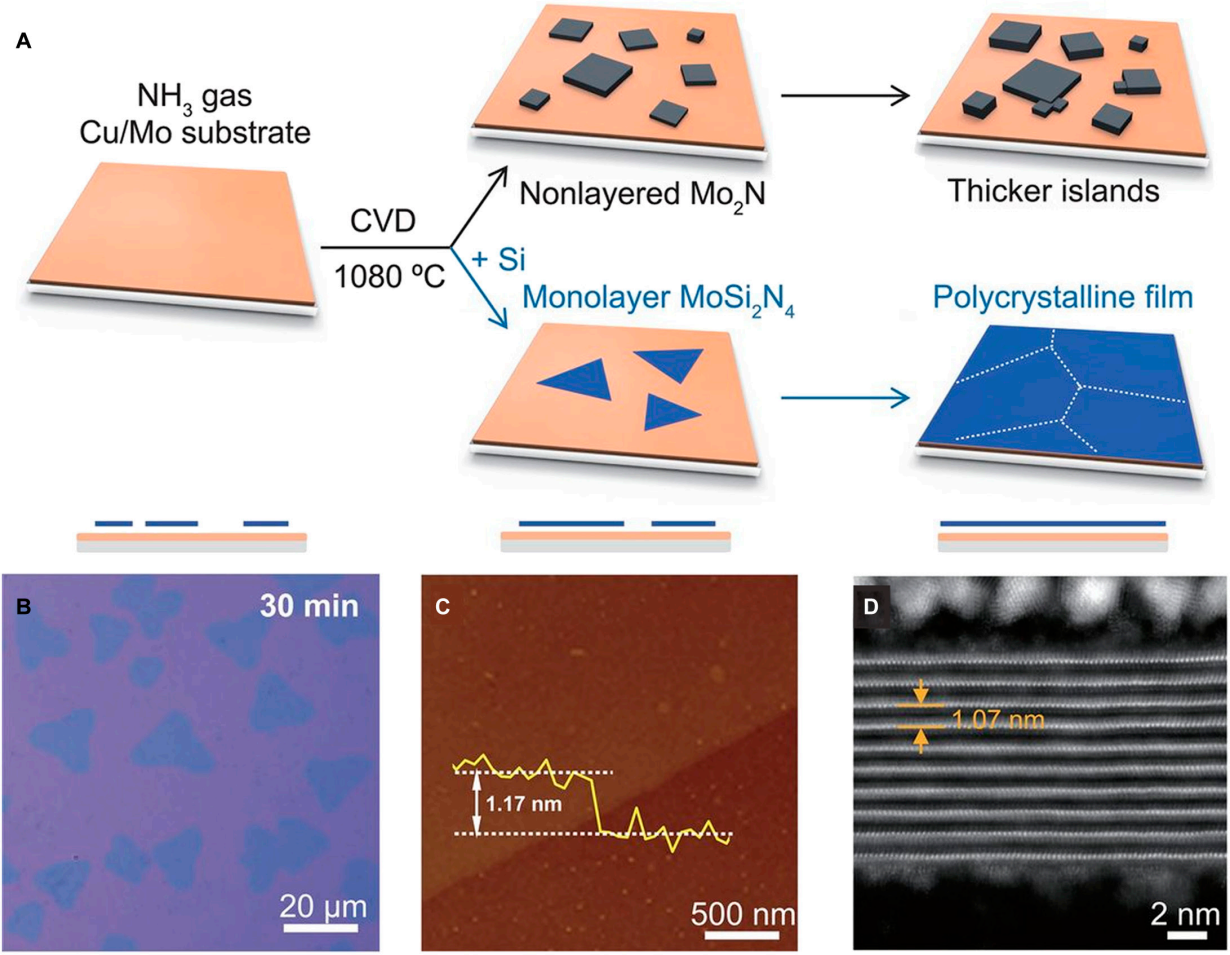
CVD growth of MoSi_2_N_4_. (A) Schematic of growth processes to synthesize layered MoSi_2_N_4_ film. (B) Optical images of MoSi_2_N_4_ grown by CVD for 30 min, which was transferred onto SiO_2_/Si substrates. (C) A typical AFM image of MoSi_2_N_4_ film with a thickness of ~1.17 nm. (D) Cross-sectional high-angle annular dark-field (HAADF) scanning transmission electron microscopy (STEM) image of a thick layered MoSi_2_N_4_ domain with an interlayer spacing of ~1.07 nm. Reproduced with permission from Ref. [24], Copyright 2020, American Association for the Advancement of Science.

Despite complicated compositions and lattice structures for vdW ternary materials, the bottom-up strategies can be utilized to synthesize their 2D nanomaterials. Given that CVD has been widely performed to synthesize TMD monolayers, it can also be utilized to grow 2D ternary TMD alloys with random or ordered atomic substitution. By precisely controlling the kinetics and thermodynamics of growth reaction, the randomized and Janus MoSSe monolayer alloys can be synthesized [62]. As a scalable and reliable technique, CVD not only shows great promise for achieving large-area samples with controllable quality and thickness, but also produces 2D materials without known 3D parents. Hong et al. [24] introduced appropriate elements to passivate the high-energy surfaces of non-layered metal nitrides during CVD growth, which leads to the growth of a new family of vdW materials, 124-type MoSi_2_N_4_ and WSi_2_N_4_ (Fig. 9). Molecular beam epitaxy growth is also a bottom-up method to synthesize high-quality film of ternary materials [78,79]. Zhu et al. [78] have systematically studied the growth conditions and kinetics of MnBi_2_Te_4_ thin films by virtue of the molecular beam epitaxy technique. They found high growth temperature that allows the nucleation of the films is crucial to minimize the density of Mn substitutional atoms on Bi sites, thereby obtaining high-quality MnBi_2_Te_4_ films. Besides, the 111-type BiTeX (X = Cl, Br) can be synthesized by epitaxial synthesis. Through the conversion of Bi_2_Te_3_ sheets in the presence of BiX_3_ vapor, ultrathin BiTeCl and BiTeBr sheets with a thickness of less than 10 nm can be grown on sapphire substrates [80].

#### Wet chemical synthesis

As one of the bottom-up strategies, wet chemical synthesis can also be used for the preparation of 2D vdW ternary materials, especially metal oxyhalides. BiOCl nanosheets are found to be synthesized by a hydrothermal/solvothermal method, and the morphology and structure can be readily modulated by various preparation parameters, such as reaction time, temperature, pressure, and concentration of surfactants [81–83]. Additionally, laser ablation in liquid solution is developed to synthesize the crystalline FeOCl nanosheets at ambient conditions, and the crystalline size and proportion of FeOCl can be effectively modulated by altering FeCl_3_ concentrations [84].

### Non-vdW ternary layered materials (112-type, 221-type, and MAX phase)

Non-vdW ternary materials, such as 112-type and 221-type compounds or MAX phases, are a group of materials constructed by chemical bonds in all 3 dimensions. Recently, both experimental results and theoretical calculations proved that thinning non-vdW solids down to 2D morphology, such as metal nitrides, hematite, and silicon, can lead to the remarkable change of intrinsic properties including bandgap opening, high room-temperature electron mobility, and 2D ferromagnetism [85–88]. Moreover, 2D non-vdW materials usually produce rich tangling chemical bonds on the surface, which could allow covalent functionalization with different termination groups and thus expand the applications in electronics, magnetism, and energy storage. Since non-vdW ternary layered materials contain a large amount of materials with diverse physical and chemical properties, it is important to investigate non-vdW ternary layered materials with the thickness down to the 2D limit. However, the complicated compositions and strong chemical bonds between neighboring layers in non-vdW ternary materials make it more challenging to exfoliate them into 2D structures than the vdW layered materials and simple non-vdW compounds. Considering that the chemical reactivity and bond strength are discriminative for the chemical bonds between different layers, deliberately constructing a gap layer to selectively weaken the interlayer interaction is proposed to achieve effective exfoliation of non-vdW ternary materials. For instance, the M–A bonds are more chemically active than the M–X bonds in MAX phase, and A atoms can be selectively extracted using highly reactive solvents, such as hydrogen fluoride and strong acids, which leads to effective production of few-layer-thick 2D transitional metal carbides and nitrides. In order to selectively remove chemical-active layers, several etching routes have been proposed.

#### Wet chemical etching and exfoliation

**Fig. 10.**
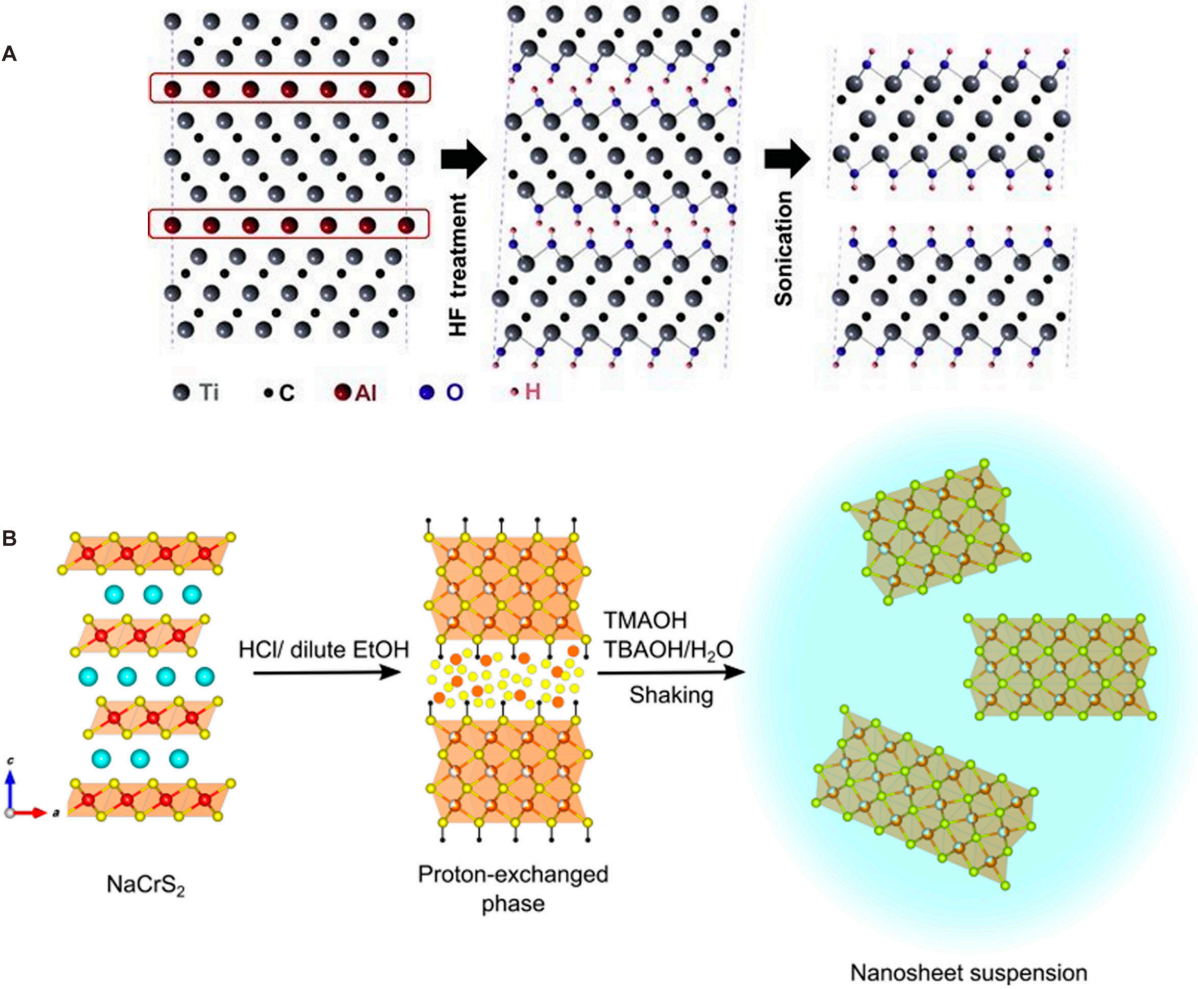
Schematic of 2 routes for wet chemical exfoliation of non-vdW ternary materials. (A) Chemical etching by an F-containing solution to produce MXene nanosheets. (B) Soft chemical process for exfoliating NaCrS_2_ into nanosheet suspension. Panel (A) is reproduced with permission from Ref. [56], Copyright 2011, Wiley-VCH. Panel (B) is reproduced with permission from Ref. [91], Copyright 2019, American Chemical Society.

Wet chemical processes could allow a high-yield, low-cost, and massive production of ultrathin 2D nanosheets in a solution phase. As one of the wet chemical etching strategies, soft chemical reactions have been shown to open an interlayer gap and achieve efficient exfoliation in 112-type compounds. Generally, a soft chemical process has been established to exfoliate many non-vdW layered materials through the following steps: initial interlayer ion exchange, subsequent lattice swelling, and exfoliation with the aid of shearing force in a solution. For example, the production of oxide nanosheets is derived from compounds consisting of layered slabs of corner- or edge-shared MO_6_ octahedra (where M = Ti, Mn, Nb, etc.) and interlayer alkali metal cations, such as K^+^, Rb^+^, Cs^+^, etc. After being treated in an acid solution, these layered compounds can be easily converted into hydrated protonic forms. Interestingly, the protons between the metal oxide slabs can be readily exchanged with large-sized organoammonium ions, accompanied by the insertion of massive solvent molecules aiding the subsequent exfoliation process [89]. Soft chemical etching can also be utilized in AMX_2_ compounds, such as LiCoO_2_ and NaCrS_2_ [90–92]. Schoop’s group treated NaCrS_2_ crystals with a HCl/ethanol solution with a proton exchange reaction to obtain a H*_x_*CrS_2_-base layered material, which can subsequently be treated with an aqueous alkylammonium solution to synthesize H*_x_*CrS_2_ nanosheet suspension (Fig. 10B). Through this wet chemical method, a completely new material, H*_x_*CrS_2_ nanosheet, has been developed, and its enhanced magnetic frustration property has been explored [91].

Etching active layers via treatment with an F-containing solution is another general wet chemical recipe to build gap layers in non-vdW ternary solids, especially MAX phase materials. In 2011, Naguib et al. [56] first reported the exfoliation of Ti_3_AlC_2_ by treatment in a hydrofluoric acid solution. As demonstrated in Fig. 10A, after the immersion in the hydrofluoric acid (HF) solution, individual layers are clearly separated from each other, readily harvesting with Ti_3_C_2_T*_n_* nanosheets terminated by hydroxyl or fluorine groups (which are denoted as T groups) with the assistance of sonication [93]. The removal of the A-site layers of MAX phases results in the 2D MXenes, which are labeled due to their structural similarities with graphene. Later, the selective etching of the A-layer in MAX phases has been developed with the aid of other aqueous solutions containing fluoride ions, such as ammonium bifluoride ((NH_4_) HF_2_) [94]. Interestingly, through high-throughput computational calculation, it is predicted that only MAX phases can be exfoliated to 2D MXenes when their M–A bonds are weaker than the M–X bonds [95].

#### Thermal etching and exfoliation

Thermal etching is an effective method to construct gap layers in non-vdW materials to achieve exfoliation. Recently, Li et al. [96,97] reported a generic method to put MAX phases into Lewis acidic molten salt at high temperatures so as to etch the A-site elements by direct redox reaction, which drastically increases the production efficiency of MXenes. Taking Ti_3_SiC_2_ as an example, the controlled redox strategy involves the following reactions with CuCl_2_ at 750 °C:

$${\text{Ti}}_{3}\text{Si}C_{2}+2\text{Cu}{\text{Cl}}_{2}\to {\mathrm{Ti}}_{3}C_{2}+\mathrm{Si}C_{2}+\text{Si}{\text{Cl}}_{4}\left(g\right)+2\mathrm{Cu}$$

$${\text{Ti}}_{3}\text{Si}C_{2}+\text{Cu}{\text{Cl}}_{2}\to {\text{Ti}}_{3}C_{2}{\text{Cl}}_{2}+\text{Cu}$$

**Fig. 11.**
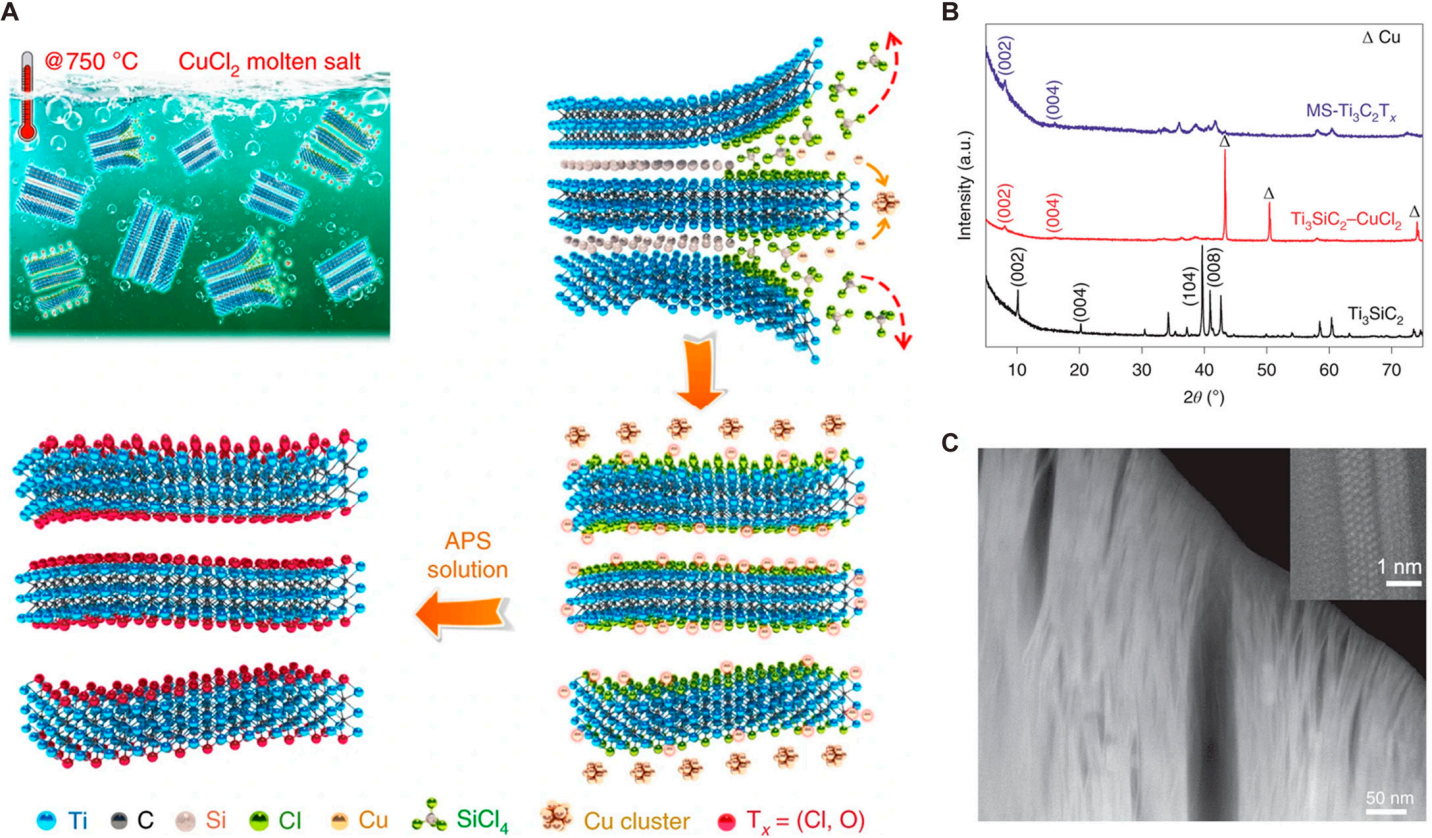
A general Lewis acidic etching route for MXene preparation. (A) Schematic for preparing Ti_3_C_2_T*_x_* MXene. (B) XRD patterns of pristine Ti_3_SiC_2_ (black line), reaction with CuCl_2_ (red line), and final MS-Ti_3_C_2_T*_x_* MXene (purple line). (C) Cross-sectional HAADF-STEM image of the MS-Ti_3_C_2_T*_x_* MXene. Reproduced with permission from Ref. [96], Copyright 2020, Nature Publishing Group.

In this procedure, the weak bonds between Si atoms and Ti in the Ti_3_SiC_2_ sublayers play a critically important role in the formation of volatile SiCl_4_. It is interesting that the gas products formed in the etching process can act as an effective expansive agent to exfoliate the MXene. As shown in Fig. 11, the obtained TiC_2_T*_x_* MXene exhibits a distinct lamellar microstructure. Note that the formation mechanism of MXene from the parent MAX phase by thermal etching is analogous to that of chemical etching in an HF solution. Besides MXene, transition metal chalcogenides with substituted heteroatoms can also be synthesized from non-vdW bulk solids by the approach of chemical etching. Du et al. [98] found that the M–A bonds in MAX phases can react easily with chalcogen-containing gases at high temperatures, which results in a product of AZ and MZ materials. Most importantly, the gaseous state of AZ intermediate products at high vapor pressure would evaporate quickly and further boost a continuous reaction and open a vdW gap between the layers. The atomic-resolution STEM image of the as-obtained TiSe_2_ further reveals its phase structure with 1T coordination. Based on this principle, 13 kinds of 2D TMD vdW structures have been synthesized from non-vdW MAX phases. Besides, Guo et al. [85] have developed a CaH_2_-assisted chemical etching method to selectively remove the Cl atoms of ZrNCl and obtain a vdW-like ZrN layered crystal. The layered structure can thus be exfoliated into highly oriented and stable cubic ZrN nanosheets. Strikingly, the vdW-ZrN layers exhibit distinct confined dimensional effects with the 2D superconducting behavior of the unconventional upper critical field far beyond the Pauli paramagnetic limit. Compared to wet chemical etching, the thermal etching route can achieve high-crystallinity 2D materials and well-defined termination of layers, which can be used to explore the emerging novel electronic properties.

#### Electrochemical etching and exfoliation

**Fig. 12.**
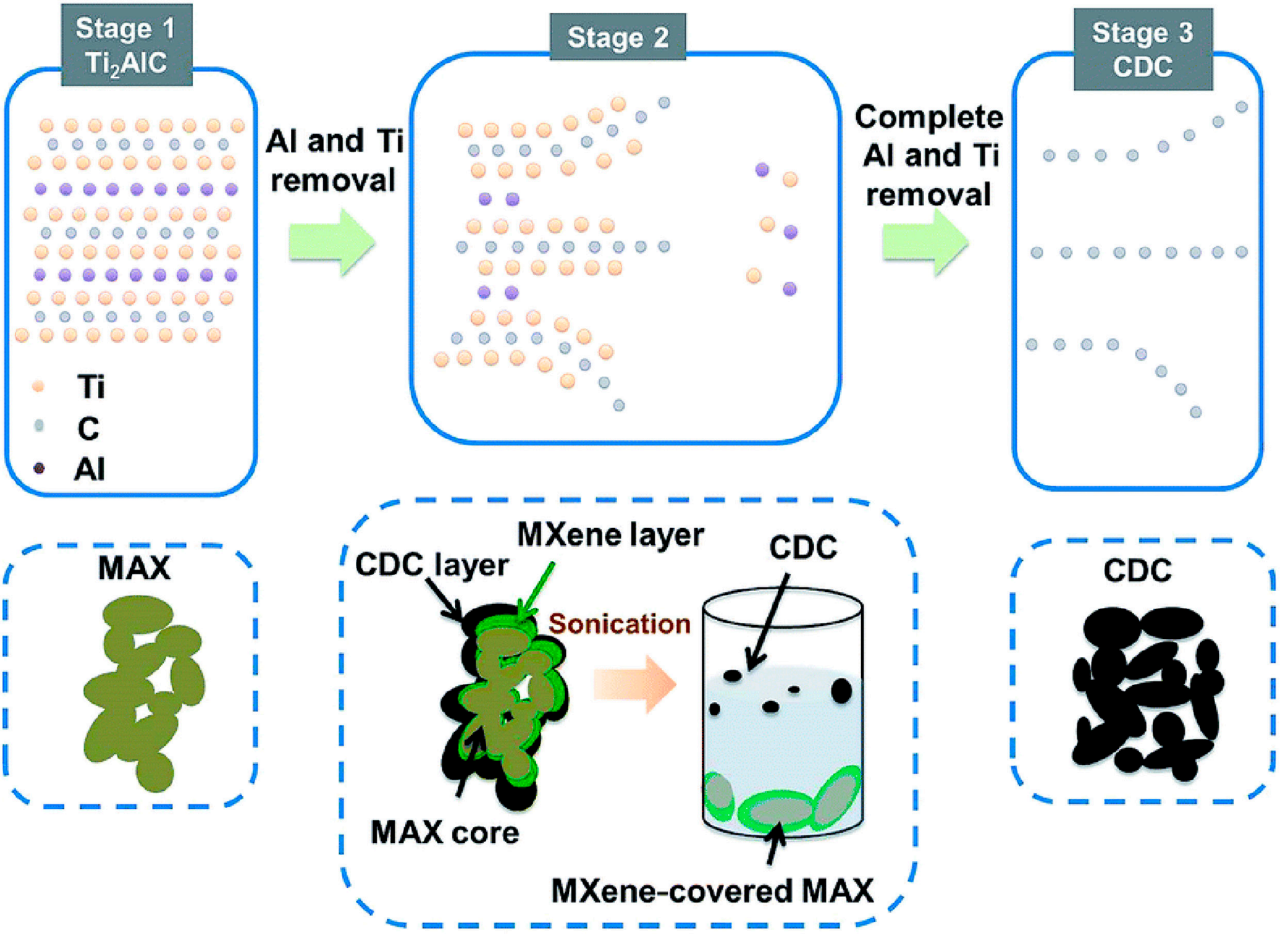
Schematic of electrochemical etching of Ti_2_AlC in a HCl aqueous electrolyte. Reproduced with permission from Ref. [101], Copyright 2017, The Royal Society of Chemistry.

Electrochemical intercalation, a process occurring on some metal ion batteries when an anode is discharged or a cathode is charged, has been proven powerful in material exfoliation. Typically, electrochemical intercalation can easily introduce guest ion insertion in vdW solids, such as graphite and TMDs, which leads to lattice expansion and further exfoliation [43,99]. However, in non-vdW solids, a de-intercalation step should be introduced before exfoliation. During the electrochemical process, chemically active layers are reduced/oxidized and removed when the bulk is set in a cathode/anode. Simultaneously, large-sized ions and solvent molecules are inserted into the empty layers and expand the interlayer distance. Recently, Peng et al. [39] have synthesized ternary chalcogenide nanosheets from a non-vdW AMX_2_ crystal by electrochemical etching and exfoliation. The redox potential difference strategy was utilized to reduce the A-site metal ions into the elemental state, with tetraalkylammonium (TAA^+^) ions being intercalated because of their lower redox potential than that of the A-site ions. Except for 112-type compounds, the MAX phase can also be electrochemically etched and exfoliated into MXene [101,101]. Porous Ti_2_AlC electrodes were reported to be exfoliated into Ti_2_CT*_x_* MXenes by means of electrochemical etching of Al in dilute hydrochloric acid, and Ti was simultaneously removed with Al if the etching time is prolonged (Fig. 12). Prominently, the adjustable working voltage and time in electrochemical etching can precisely regulate the extraction of chemically active layers, which can be used to control the chemical components of the resultant 2D nanomaterials.

#### Chemical vapor deposition

**Fig. 13.**
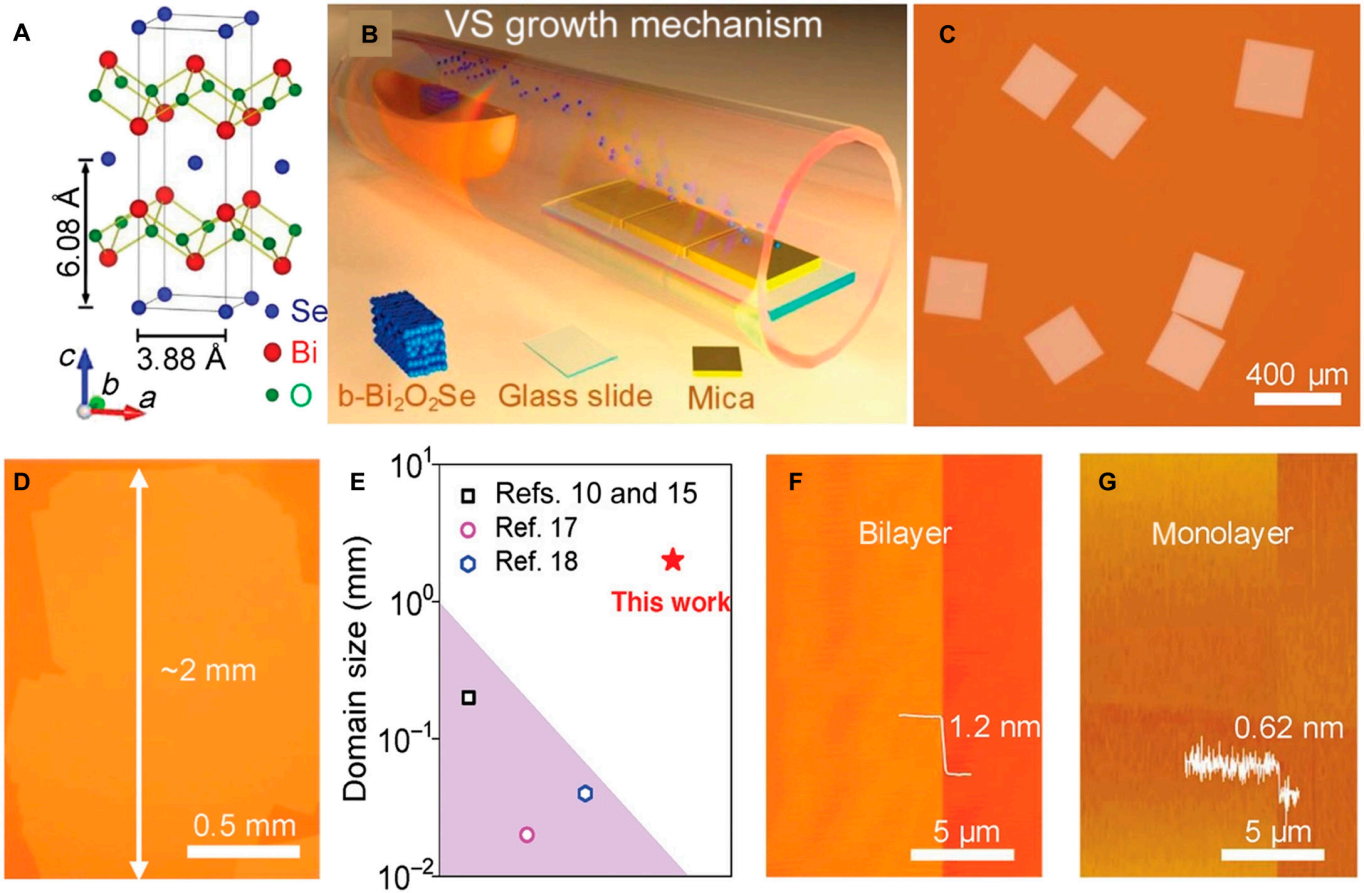
Vapor-solid growth of 2D Bi_2_O_2_Se single crystals on mica. (A) Crystal structure of Bi_2_O_2_Se. (B) Schematic for the VS growth process of 2D Bi_2_O_2_Se. (C) A typical optical image of 2D Bi_2_O_2_Se single crystals. (D) An optical image of millimeter-sized 2D Bi_2_O_2_Se. (E) Domain sizes of 2D Bi_2_O_2_Se reported in the literature. (F and G) AFM image of bilayer and monolayer Bi_2_O_2_Se flakes, respectively. Reproduced with permission from Ref. [102], Copyright 2019 The Wiley-VCH.

Considering the complicated compositions and the types of interlayer bonding in non-vdW ternary materials, it is more challenging to grow their 2D materials with well-defined chemical stoichiometry and high crystal quality than that of graphene and TMDs. The strong interlayer interactions suggest high surface energies of 2D ternary materials, which usually results in easy vertical growth into bulk crystals. To date, only several non-vdW ternary materials can be synthesized with 2D form through a bottom-up approach [17,102–104], and the source concentrations of 3 elements should be carefully controlled since they remarkably affect the domain size and thickness of as-grown 2D samples. For 221-type compounds, the CVD route is an efficient way to synthesize their 2D counterparts. It has been reported that the concentration and pressure of O_2_ are crucial to the lateral size of Bi_2_O_2_Se nanosheets. In order to achieve Bi_2_O_2_Se nanosheets with a large domain size, we proposed a self-limiting vapor–solid deposition approach to grow Bi_2_O_2_Se on a mica substrate at ambient pressure [102] (Fig. 13A to C). Because of the lack of suitable adsorption sites, the precursor molecules prefer to absorb at the edges of the newly grown Bi_2_O_2_Se flakes rather than at the top surfaces, leading to the growth of atomically thin materials with a millimeter domain size (Fig. 13D to G).

### The composition and structure of 2D ternary layered materials

It is known that the chemical compositions and lattice structures determine the electronic structure and properties of materials. Therefore, controllable synthesis becomes of great importance to explore the intrinsic properties of ternary layered materials with atomic thicknesses. It is worth noting that the composition and structure of as-exfoliated vdW ternary nanosheets can be well maintained with their parent bulk. This consistency can be attributed to the weak interlayer interaction and saturated layer surface in vdW layered materials. On the contrary, non-vdW ternary materials show no obvious gap between interlayers, which demands chemical etching to remove or replace some atoms and thus construct a vdW gap. As a result, 2D materials derived from non-vdW ternary materials usually undergo drastic changes either in composition or structure. Moreover, there should be numerous unsaturated atoms and dangling bonds on the surface of 2D materials after being thinned down from non-vdW solids, making it unavoidable to adsorb or even bond with other ions or molecules so as to compensate for the charge. Interestingly, these dangling bonds may provide an opportunity to modify the 2D materials with certain functional groups or atoms, which greatly alters their physical and chemical properties and even creates new materials. In this section, we summarize the modulation of chemical compositions and surface functional groups in 2D ternary layered materials.

#### Chemical composition

The rational design and engineering in composition and structure of 2D materials are important for developing diverse applications. As for vdW ternary materials, their compositions and structures are preserved after exfoliation. Thus, a practical and facile route to modify 2D vdW ternary materials is by directly modifying the precursor crystals through element substitution or doping [105,106]. For instance, 2D high-entropy MPX_3_ can be produced through conventional chemical vapor transport followed by sonication-assisted exfoliation. Compared to CoPS_3_, the Co_0.6_(VMnNiZn)_0.4_PS_3_ nanosheets exhibit enhanced hydrogen evolution reaction (HER) performance [105]. In the case of non-vdW 2D materials, the exfoliation process typically requires the etching of active atoms to form gap layers, which would result in the change of chemical composition compared to their parent bulk crystals. For example, MXene nanosheets are derived from MAX phases by selectively etching the A-site atoms leaving with the 2D carbide and nitride layers [55]. The MXenes are usually terminated with anions, commonly denoted as M_*n*+1_X*_n_*T*_x_*, so as to neutralize the materials. Similarly, another category of typical non-vdW ternary layered solids, 112-type AMX_2_ compounds (where A is a monovalent metal, M is a trivalent metal, and X is a chalcogen), can be exfoliated into 2D structures with changed chemical composition. NaCrS_2_ crystals were exfoliated into H_x_CrS_2_ nanosheets through proton exchange in a HCl/ethanol solution [91].

**Fig. 14.**
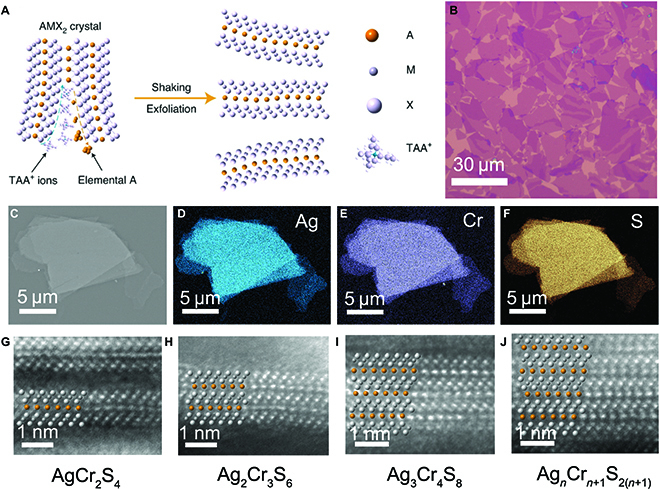
Synthesis of stoichiometric non-vdW AgCrS_2_ nanosheets. (A) Schematic for the intercalation and exfoliation of the AMX_2_ crystals. (B) Optical image of as-exfoliated AgCrS_2_ nanosheets. (C to F) SEM image and corresponding elemental mapping images of an exfoliated AgCrS_2_ nanosheet by EPMA analysis. (G to J) Cross-section HAADF-STEM images of a single-layer (G), bilayer (H), trilayer (I), and multilayer (J) AgCrS_2_ nanosheet. Reproduced with permission from Ref. [39], Copyright 2021, Nature Publishing Group.

Recently, in order to preserve interlayer A-site atoms from entirely etching, we developed a redox-controlled strategy to exfoliate AgCrS_2_ crystal into a similar composition and structure to those of the bulk materials [39] (Fig. 14). By taking advantage of the continuously adjustable working voltage and large radius of TAA^+^ ions, the exfoliated large-sized nanosheets after electrochemical etching are stoichiometric 2D non-vdW materials with the formula Ag*_n_*Cr_*n*+1_S_2(*n*+1)_ even in monolayer. In this case, the electrochemical exfoliation process exhibits precise control of the etching degree and avoids the substantial sacrifice of the interlayer of active atoms, successfully maintaining the composition of the exfoliated layers coherent with the parent bulk materials.

#### Surface termination

**Fig. 15.**
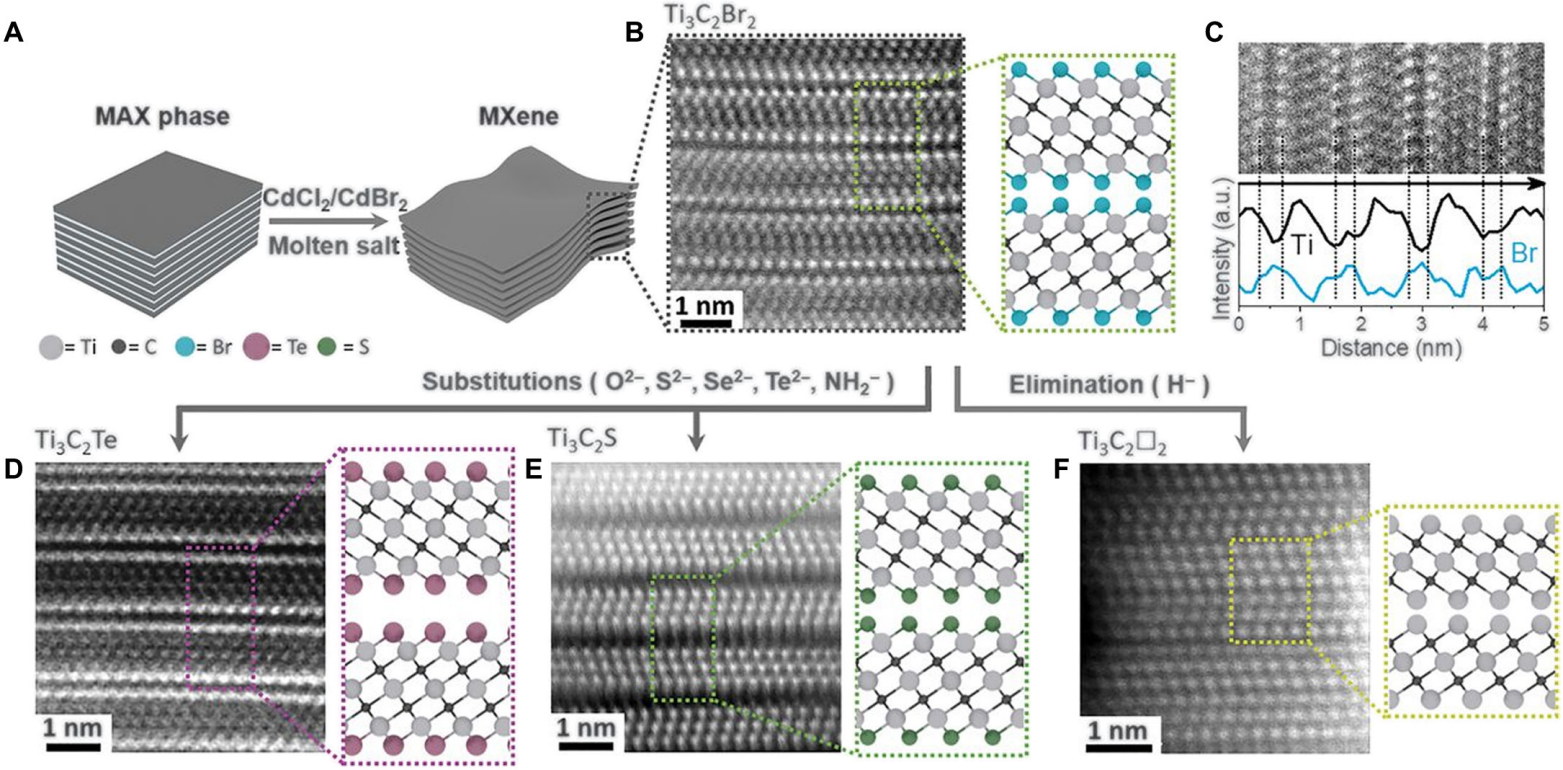
Covalent surface modifications of MXenes. (A) Schematics for etching of MAX phases in Lewis acidic molten salts. (B and C) HAADF-STEM image and elemental analysis of Ti_3_C_2_Br_2_ MXene sheets. (D to F) HAADF-STEM images of Ti_3_C_2_Te, Ti_3_C_2_S, and Ti_3_C_2_□_2_ MXenes ( □ represents the vacancy). All scale bars are 1 nm. Reproduced with permission from Ref. [22], Copyright 2020, American Association for the Advancement of Science.

In fact, the physical and chemical properties of 2D materials with the atomic thickness could be largely influenced by their surface due to the confinement effect. Many appealing applications of 2D ternary materials, including electronics and energy conversion and storage, call for the materials with certain surface functional groups. In addition, the exfoliated non-vdW 2D ternary materials possess numerous dangling bonds, which can serve as an ideal platform to install or remove surface groups so as to realize the rational design and engineering in material composition or structure. Theoretical calculations predicted that selectively changing the termination of MXenes with different atoms can result in a remarkable change in band structure [107,108]. Specifically, M_2_C (M = Sc, Ti, Zr, Hf) undergoes a transition from metal to semiconductor when terminated with F, OH, or O groups. Recently, Kamysbayev et al. [22] reported the covalent surface modification of MXenes. They synthesized MXenes terminated with O, NH, S, Cl, Se, Br, and Te atoms, and comprehensively investigated the structure and property changes (Fig. 15). In detail, MXene such as Nb_2_C with S, Se, and NH termination exhibits superconductivity, whereas the macroscopic superconducting behavior is suppressed in CO*_x–_* terminated ones due to the weak coupling of domains. Therefore, engineering of the chemical bonds in a non-vdW stack can be expected to achieve material engineering with brand-new properties and applications.

## Properties and Applications

Similar to graphene and MoS_2_, ternary layered materials with atomic thicknesses exhibit many different electronic properties from bulk parents because of the quantum confinement effect. Such ternary materials with diverse chemical compositions can be metals, semiconductors, and insulators, which shows a wide spectrum of exotic behaviors that have not yet been explored. In addition, the as-produced 2D materials have been shown to be compelling in electronics, optoelectronics, and electrochemistry.

### Layer-dependent properties

Owing to the strong anisotropy in ternary layered materials, they can be exfoliated in a layer-by-layer manner. Thus, we can investigate their layer-dependent features, which is essential for an in-depth understanding of the variation of the electronic structure under the confinement effect. vdW ternary layered materials can keep the chemical composition and stoichiometric ratio unchanged as the thickness is reduced down to monolayer. However, for non-vdW ternary solids, the study of intrinsic properties of their 2D counterparts suffers from the dramatic alteration of composition and structure during the exfoliation process. Therefore, the layer-dependent properties are summarized mainly toward vdW ternary materials.

**Fig. 16.**
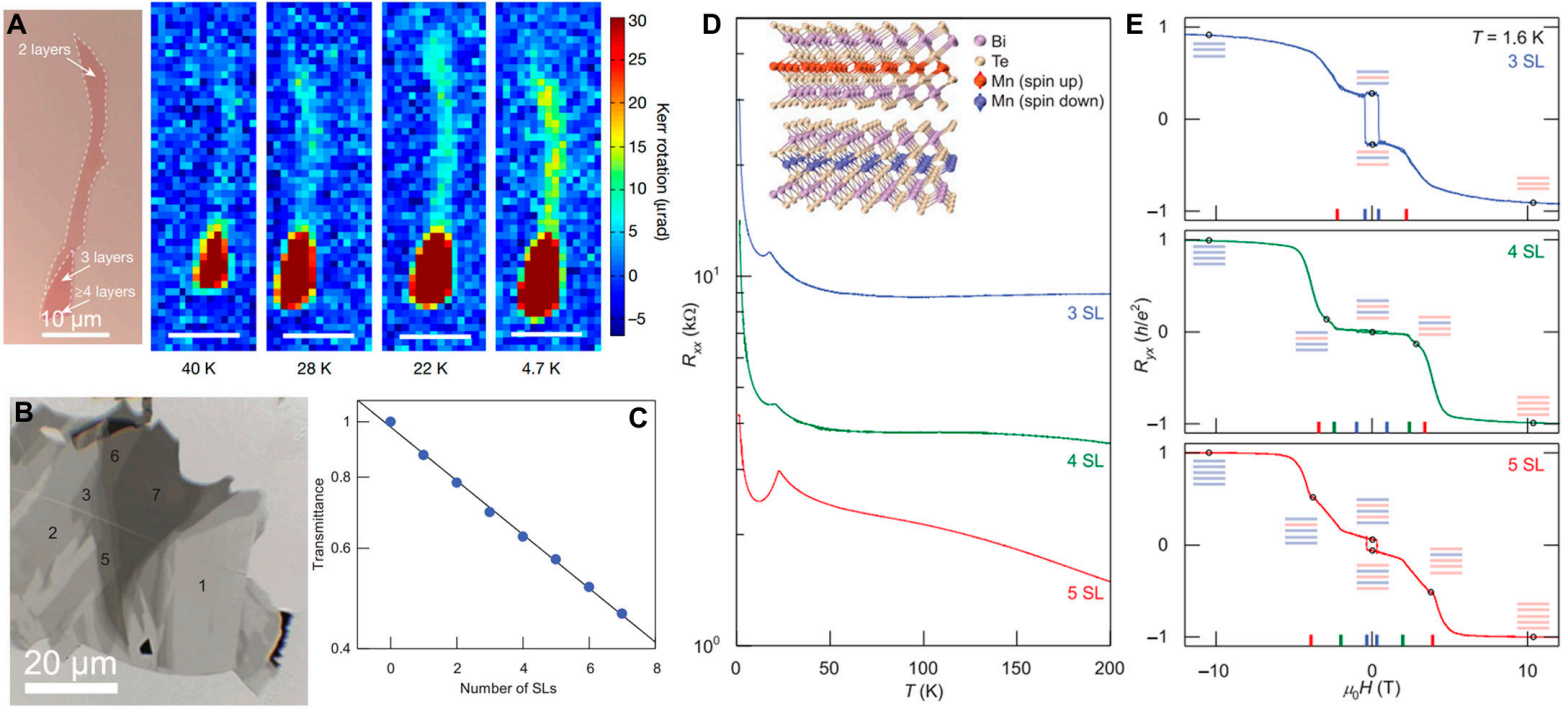
The magnetism and quantum anomalous Hall effect of 2D ternary layered materials. (A) Observation of ferromagnetism and Kerr rotation signal in bilayer Cr_2_Ge_2_Te_6_. (B) Optical image of few-layer flakes of MnBi_2_Te_4_ and (C) transmittance as a function of the number of single layers. (D) Temperature-dependent sample resistance of few-layer MnBi_2_Te_4_. (E) *R_yx_* of the same MnBi_2_Te_4_ samples with different thicknesses shown in panel (B). Part of panel (A) is reproduced with permission from Ref. [49], Copyright 2017, Nature Publishing Group. Parts of panels (B) to (E) are reproduced with permission from Ref. [19], Copyright 2020, American Association for the Advancement of Science.

There have been a large amount of theoretical and experimental results proving that the electronic, optical, and spin structures change substantially, as the ternary layered materials are reduced to the 2D limit. The calculation results show that the electronic bandgap of 111-type BiTeCl and BiTeBr increases as the number of layers decreases, and a monolayer becomes semiconducting with bandgaps of 0.83 and 0.80 eV, respectively [109]. In addition, monolayer ZrNCl is predicted to have a larger bandgap than few-layer and bulk materials, and Nong et al. [63] found the layer-dependent Raman scattering with a blueshift of out-of-plane A_1g_ peak in atomic thick samples. Prominently, the A_1g_ peak is absent in single-layer ZrNCl, which suggests that Raman spectroscopy can be a tool to identify the number of layers. Indeed, Raman spectroscopy is a rapid and nondestructive route to study layer-dependent properties of 2D materials, and it has been utilized to investigate the spin dynamics of 113-type compounds as thickness is thinned down. It is found that many 113-type ternary materials possess intrinsic magnetic properties even surviving in monolayer. For example, a magnetic persistence in FePS_3_ even down to monolayer regime is observed through layer-number-dependent Raman spectra, which points out that the Néel temperature decreases from 117 K in bulk to 104 K in the monolayer counterpart [16]. Except for antiferromagnetic FePS_3_, the layer-dependent magnetism can also be uncovered in ferromagnetic Cr_2_Ge_2_Te_6_. Gong et al. [49] observed a strong dimensionality effect in Cr_2_Ge_2_Te_6_ flakes with different thicknesses through temperature-dependent Kerr rotation measurements. As can be seen in Fig. 16A, a monotonic decrement of transition temperature is revealed with decreased thickness, where the value reduced from 68 K in bulk to about 30 K for a bilayer. The strong thickness dependence of magnetism demonstrates that interlayer magnetic coupling is essential in establishing the ferromagnetic order for the ternary Cr_2_Ge_2_Te_6_.

Compared to the monotonic variation of magnetic properties in 113-type compounds, the 124-type MnBi_2_Te_4_ is endowed with an intrinsic magnetism depending on the even or odd number of layers [19,110]. Each septuple layer of MnBi_2_Te_4_ is ferromagnetically coupled within the in-plane Mn atomic layers, but antiferromagnetically between adjacent septuple layers. As a result, the top and bottom surface magnetizations are antiparallel or parallel according to the layer number of MnBi_2_Te_4_ flakes (Fig. 16B to E), and the Hall conductance from the 2 surfaces may cancel or sum, which leads to a clear even–odd layer-dependent anomalous Hall effect.

Similar to vdW layered materials, the electronic structure and properties of non-vdW ternary materials also reveal a function against layer number, especially when the chemical composition and structure of as-exfoliated nanosheets are preserved from bulk. Through the first-principles calculation, Zhong et al. [111] demonstrated that the ferromagnetism can be greatly stabilized above room temperature in thin-layer CuCrS_2_ and CuCrSe_2_ compared with their bulk phases, which show non-vdW layered structures with antiferromagnetism. Furthermore, we have experimentally achieved AgCrS_2_ nanosheets with Ag layer integrally preserved within CrS_2_ layers. Intriguingly, the ionic conductivity of individual AgCrS_2_ flakes monotonically increases as the layer number decreases, and the monolayer sample shows superionic behavior at room temperature, while in the bulk AgCrS_2_, the behavior can only be observed above 400 °C [39].

### Electronic/optoelectronic devices

**Fig. 17.**
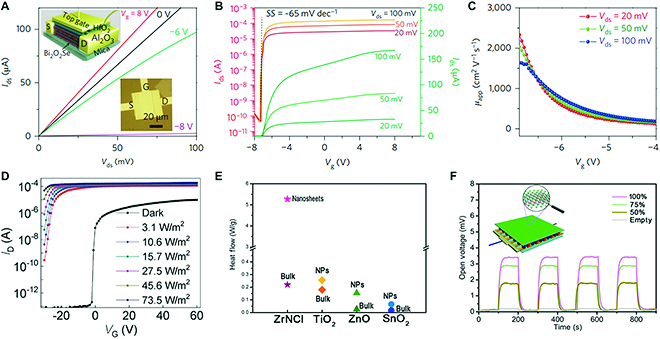
The electronic and optoelectronic performance of 2D ternary layered materials. (A and B) Output curves and top-gate transfer curves of top-gated Bi_2_O_2_Se-channel FETs. (C) Field-effect mobility as a function of gate voltage. (D) Gate-tunable photoresponse of 2D Bi_2_O_2_Se phototransistors. (E and F) Photothermal effect of ZrNCl film assembled by nanosheets. Panels (A) to (C) are reproduced with permission from Ref. [17], Copyright 2017, Nature Publishing Group. Panel (D) is reproduced with permission from Ref. [102], Copyright 2019, The Wiley-VCH. Panels (E) and (F) are reproduced with permission from Ref. [73], Copyright 2015, American Chemical Society.

Ultrathin 2D materials with single and few layers show intriguing mechanical and electronic properties, which offer an ideal candidate to form a basis for next-generation electronic device geometries. Thus, ternary layered materials with the 2D structure show great promise in nanoelectronic research including field-effect transistors (FETs), photodetectors, and spin-related devices. Many 111- and 124-type compounds are semiconducting with a suitable bandgap, excellent chemical and thermal stability, and high theoretical mobility. Monolayer MoSi_2_N_4_ is predicted to have intrinsic electron and hole mobilities of ~270 cm^2^ V^−1^ s^−1^ and ~1,200 cm^2^ V^−1^ s^−1^ at K point in the Brillouin zone, respectively [24]. Experimentally, back-gated FETs with monolayer MoSi_2_N_4_ flakes were measured to evaluate their electrical transport properties. The devices show good stability in air and vacuum and possess typical p-type semiconductor behavior with an on/off ratio reaching 4,000 at 77 K, while the measured mobility was inferior to the theoretical data. Similarly, trilayer ZrNCl samples have been measured to show a high on/off ratio of 10^8^, but the maximum electron mobility is only 3.15 cm^2^ V^−1^ s^−1^ when served as channel materials in FET devices [63]. In order to realize evident regulation of electronic phase for 2D ternary materials, electric double-layer transistors were fabricated using ionic liquid as gate dielectrics, with a micro-cleaved semiconducting ZrNCl as the channel material [64,112]. As the gate voltage increases, the resistance of the ZrNCl flake produces a remarkable reduction, and insulator-metal transition occurs. Prominently, when the gate voltage increases to more than 3.5 V, superconductivity was induced in the ZrNCl flake with a transition temperature of 15.2 K [64]. Besides vdW ternary materials, non-vdW Bi_2_O_2_Se nanosheets have recently attracted tremendous interest due to their air stability and superior electronic performance. It is found that the electron Hall mobility value of as-grown Bi_2_O_2_Se nanoflakes can reach up to 20,000 cm^2^ V^−1^ s^−1^ at 1.9 K [17]. High-performance FETs can thus be fabricated based on bilayer Bi_2_O_2_Se crystals, which exhibit a high Hall mobility of 450 cm^2^ V^−1^ s^−1^, superior current on/off ratio of more than 10^6^, and near-ideal subthreshold swing values of ~65 mV dec^−1^ at room temperature (Fig. 17A to C). These results indicate that 2D ternary layered materials are promising candidates for future high-speed and low-power electronic applications.

For semiconducting ternary layered materials with a suitable bandgap, photons with energy greater than the bandgap energy can be readily absorbed or emitted, thereby providing great interest for applications in optoelectronics. MPX_3_ has an intrinsic bandgap up to 3.5 eV, suggesting excellent candidates for ultraviolet (UV) photodetectors. Indeed, it is reported that the responsivity of a FePS_3_-based UV photodetector can reach as high as 171.6 mA W^−1^ under the incident light of 254 nm [113]. On the other hand, 2D Bi_2_O_2_X with a bandgap of 0.8 eV shows ultrafast response and is highly sensitive to infrared. Concretely, high-performing infrared photodetectors based on air-stable 2D Bi_2_O_2_Se crystals exhibit a high sensitivity of 65 A W^−1^ at 1,200 nm and an ultrafast photoresponse of only 1 ps at room temperature [102] (Fig. 17D). In addition, 2D ternary layered materials can strongly interact with light. Feng et al. [73] highlighted a greatly enhanced electron–phonon interaction in 2D ZrNCl to realize giant photothermal effects (Fig. 17E and F). They found that the ZrNCl nanosheets with less than 4 monolayers can generate a heat flow of 5.25 W/g under UV irradiation, which is dozens of times higher than that of other wide-gap semiconductors.

Considering that many ternary layered materials exhibit diverse and robust magnetism when exfoliated to the 2D structure, they show great potential for integration in spintronic devices. Ostwal et al. [114] constructed Cr_2_Ge_2_Te_6_/tantalum heterostructures to manipulate the magnetization in Cr_2_Ge_2_Te_6_ thin flakes. They demonstrated that a charge current density of only 5 × 10^5^ A cm^−2^ is sufficient to switch the out-of-plane magnetization of Cr_2_Ge_2_Te_6_ under the in-plane field of 20 mT, of which the current densities are about 2 orders of magnitude lower than those required for spin-orbit torque switching nonlayered metallic ferromagnets such as CoFeB.

### Energy storage

Many 2D ternary layered materials have high electrical conductivity, processability, and stability; thus, they show superiority in energy storage devices such as batteries and supercapacitors. Although the chemical composition and lattice structure are changed dramatically compared to bulk MAX phases, the MXene nanosheets keep metal nitride/carbide layers intact, which promises ultrahigh conductivity. Theoretical calculation pointed out that the alkali ions can adsorb on MXene materials and the capacities for Li, Na, K, and Ca ions on 2D Ti_3_C_2_ can reach 447.8, 351.8, 191.8, and 319.8 mAh/g, respectively [115]. Experimentally, Li et al. [96] demonstrated a Li-ion storage capacity of up to 205 mA/g in 1 M LiPF_6_ carbonate-based electrolyte for Ti_3_C_2_ nanosheets that were produced through a Lewis acidic thermal etching route.

**Fig. 18.**
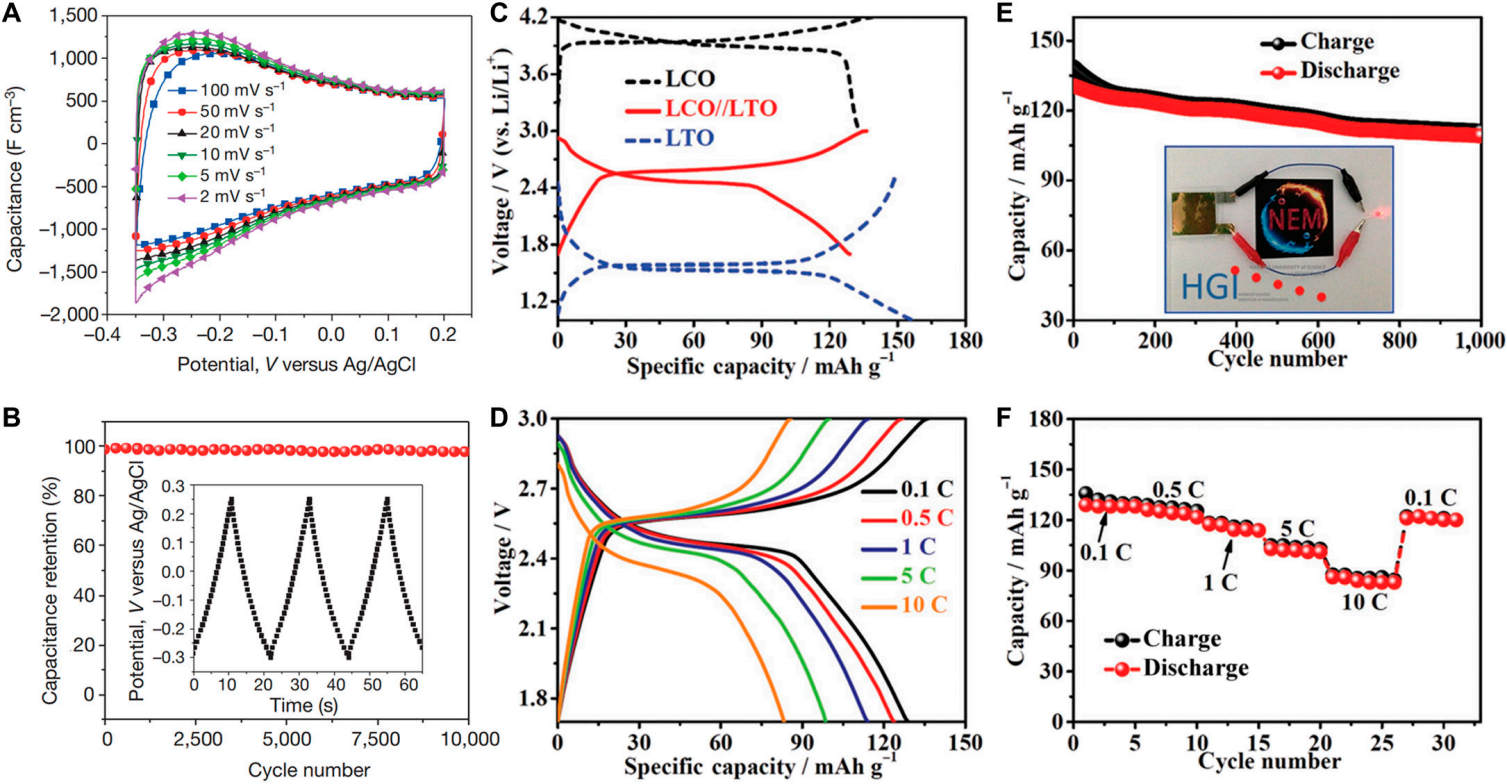
(A and B) Electrochemical capacitance performance of rolled, free-standing electrodes based on Ti_3_C_2_ nanosheets. (C) The charge/discharge curves of the LiCoO_2_ nanosheet array electrode, the Li_4_Ti_5_O_12_ nanosheet array electrode, and a LiCoO_2_//Li_4_Ti_5_O_12_ nanosheet-based full cell. (D to F) Charge/discharge curves, the cycle performance, and the rate performance of a LiCoO_2_//Li_4_Ti_5_O_12_ nanosheet-based full cell. Panels (A) and (B) are reproduced with permission from Ref. [121], Copyright 2014, Nature Publishing Group. Panels (C) to (F) are reproduced with permission from Ref. [119], Copyright 2018, The Wiley-VCH.

Furthermore, highly conductive MXene nanosheets can usually be used to serve as conducting substrates or additives, and even catalyze and accelerate the reaction in batteries for fast kinetics, leading to evident enhancement of the performance of metal-ion or lithium-sulfur batteries [116–118]. Liang et al. [116] reported a cathode consisting of 70 wt% S/Ti_2_C composites that exhibit stable long-term cycling performance with a specific capacity close to 1,200 mAh/g at a C/5 current rate, which is ascribed to the strong interaction of the polysulfide species with the surface Ti atoms. Except for MXene materials, 112-type ternary oxides can be performed as electrode materials for battery application. LiCoO_2_ is one of the most widely used cathode materials in commercial batteries. Xue et al. [119] reported that LiCoO_2_ porous nanosheet arrays grown via “hydrothermal lithiation” on spinel Co_3_O_4_ nanosheet arrays followed quick annealing. The cathode composed of LiCoO_2_ nanosheet array shows a high reversible capacity of 104.6 mAh/g at 10 C and delivers capacity retention of 81.8% at 0.1 C after 1,000 cycles (Fig. 18C to F).

Owing to high conductivity and large specific surface area, 2D ternary layered materials can also serve as electrode materials for supercapacitors, a promising energy storage device with high power density and excellent cycling life. Lukatskaya et al. [120] demonstrated that many cations can intercalate into 2D MXene layers spontaneously, and intercalation-induced high capacitances of over 300 F/cm^3^ can be achieved for flexible Ti_3_C_2_T*_x_*-based electrodes in aqueous electrolytes. When they use a solution containing lithium fluoride and hydrochloric acid to replace aqueous HF solution for etching the MAX phase, the as-exfoliated Ti_3_C_2_T*_x_* can deliver volumetric capacitances of up to 900 F/cm^3^ (Fig. 18A and B) [121]. Indeed, different etching methods result in different surface functional groups, which play essential roles in charge storage process for supercapacitor devices. It was demonstrated that more components of oxygen-contained functional groups in MXene materials can result in higher capacitance [122].

Other 2D ternary layered materials, such as 112-type oxides or sulfides, also show high performance when fabricated into supercapacitors. For example, Fukuda et al. [123] reported a 1-nm-thick H_0.2_RuO_2_∙0.5H_2_O nanosheet from wet chemical exfoliation of layered NaRuO_2_ solid. The specific capacitance of such nanosheets is comparable to that of hydrous RuO_2_ nanoparticles from sol-gel methods. Similarly, CuSbS_2_ nanoplates synthesized through solution-processing bottom-up routes can serve as electrode materials for supercapacitors [124]. The specific capacitance varies with the thickness of CuSbS_2_, and the optimized value of 120 F/g can be achieved when the thickness is about 55 nm.

### Catalysis

**Fig. 19.**
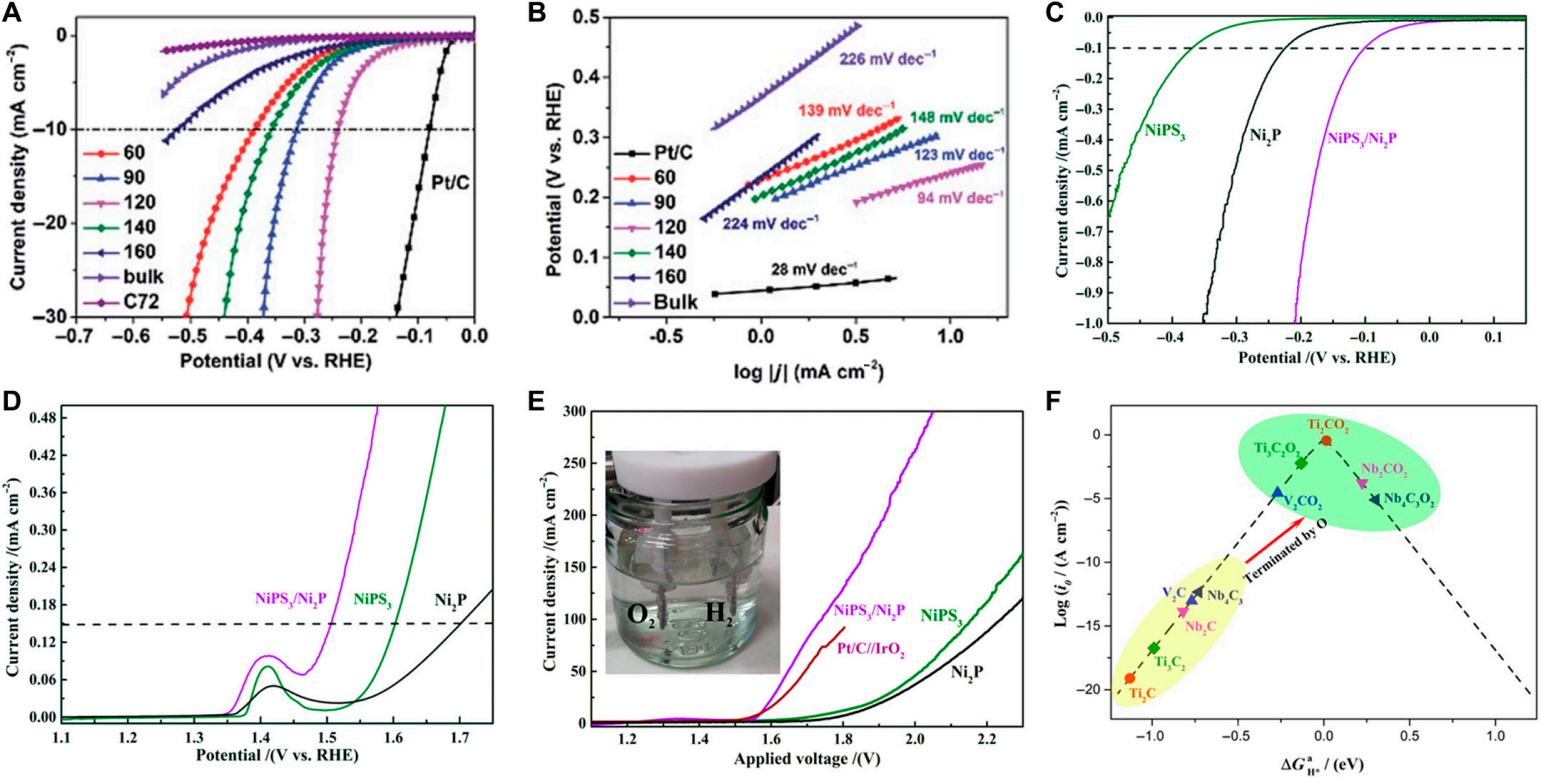
HER performance of 2D ternary layered materials. (A and B) Electrochemical measurements of the as-exfoliated FePS_3_ nanosheets in acidic media, where (A) is iR-Corrected LSV curves and (B) is Tafel plots. (C and D) LSV curves of NiPS_3_, Ni_2_P, and NiPS_3_/Ni_2_P electrocatalysts for HER and OER with normalized current density. (E) Polarization curves of water-splitting systems for the catalysts in (C) and (D). (F) Volcano curve of exchange current as a function of the average Gibbs free energy of hydrogen adsorption. Panels (A) and (B) are reproduced with permission from Ref. [77], Copyright 2019, The Royal Society of Chemistry. Panels (C) to (E) are reproduced with permission from Ref. [126], Copyright 2019, American Chemical Society. Panel (F) is reproduced with permission from Ref. [129], Copyright 2016, American Chemical Society.

Generally, 2D materials exhibit promise serving as catalysts in both electrochemical and photochemical reactions, since they possess high specific surface areas, high-performance active sites, and tunable electronic structures. In addition, many ternary layered materials are composed of non-precious metals, which show low cost for applications. Taking water electrolysis as an example, 2D ternary materials including 112- and 113-type compounds and MXene materials exhibit superior performance in hydrogen evolution or oxygen evolution reactions. MPS_3_ is found to be a good electrocatalyst for HER after being exfoliated into few-layer 2D nanosheets, and exhibiting high activity and stability over a wide pH range of electrolytes [77,125–127]. Few-layer FePS_3_ nanosheets obtained in 120 °C show mixed valence states and good crystallinity, which exhibit good acidic HER performance with an overpotential of 241 mV and a Tafel slope of 94 mV dec^−1^ (Fig. 19A and B). 2D ternary NiPS_3_ nanosheets with in-grown Ni_2_P enable an overall water splitting electrolyzer to achieve 50 mA cm^−2^ at a lower bias of 1.65 V, which is superior to that for the benchmark Pt/C//IrO_2_ electrocatalysts (Fig. 19C to E). For 2D non-vdW 112-type compounds, it is also found to be highly active catalysts for water electrolysis. The ultrathin LiCoO_2_ nanosheets exhibit a small overpotential and Tafel slope for water oxidation, with an excellent cycle life [90,128]. In recent years, MXene-based water electrocatalysts have also attracted tremendous attention because of their high conductivity and abundant active sites. Numerous experimental and theoretical researches have proven that MXene materials exhibit great potential for HER, and the performance can be further optimized through termination modifications and structural engineering [129–131] (Fig. 19F). Except for HER and oxygen evolution reaction (OER), 2D ternary compounds can also be applied in fuel cells, nitrogen fixation, and CO_2_ reduction, and achieve some preliminary progress [33,132–134].

Semiconducting ternary layered materials with a suitable bandgap can apply for photocatalysis and photo-electrocatalysis. As regards 111-type BiOX (X = halogen element), ultrathin nanosheets with facets show superior solar-driven photocatalytic activity for photodecomposition and nitrogen fixation [32,33]. On the one hand, high performance benefits from the high specific surface areas and a large number of active sites in nanosheets. On the other hand, abundant vacancies result in the effective separation of electron–hole pairs during photocatalytic reactions, thereby exhibiting an excellent ability to decompose Rhodamine B and reduce N_2_ into NH_3_.

Since MXene materials have many unsaturated dangling bonds and low-coordinated atoms, they can serve as supports to incorporate with metals to form strong interfacial metal–support interaction and show high activity in various catalytic processes [135–137]. For instance, Li et al. [137] reported that ultrathin Pt layers supported by 2D molybdenum titanium carbide nanosheets can catalyze non-oxidative coupling of methane to C2 species with high selectivity. In addition, the FeN_4_ structure supported by the highly conductive MXene can substantially promote the electrochemical activity of OER and oxygen reduction reaction [138]. Therefore, it is important to give more insights into the applications of MXene-based materials for conventional heterogeneous catalysis.

## Conclusions and Perspectives

In recent years, great effort has been made in the synthesis and exploration of ternary layered materials. This review has summarized their classification according to stoichiometry and discussed different routes to produce such 2D structure. In addition, the variation of chemical composition and atomic structure is emphasized when compared to bulk counterparts. In contrast to 2D elemental or binary materials, the complicated compositions and structures in ternary layered compounds give rise to diverse and novel members of the 2D family with exotic properties, which can thus serve as new building blocks for fantastic modifications and applications. Despite the 2D ternary layered materials exhibit great potential in many fields, standing challenges still exist and impede further exploration and applications, which requires extensive studies for this rapidly developing field. Here, we propose several key challenges and research opportunities concerning the exploration and applications of 2D ternary layered materials.

Firstly, from the material design point of view, high-throughput automation and machine learning are called for the identification of novel 2D ternary layered materials with exotic electronic structures and properties. A material database is required to be built according to the stoichiometric ratios and structural symmetries, where newly discovered or designed candidate materials should be verified and validated by experiments. Since a monolayer of ternary layered materials consists of more than 3 different atomic layers, the way for constructing their 2D structures can be renovated compared to that for binary and elemental layered materials. Specifically, they can be coupled through 2 existing binary structures with different properties, which has been proven to create 2D ternary layered solids integrated with multifunctionality [24,50]. However, currently, only a few material systems are discovered in this manner. More combination types and functional materials should be uncovered through such unique paradigm, especially the coupling of building blocks with ferroelectric, magnetic, superconducting, and topological properties. An in-depth understanding of the interlayer interaction and electronic correlation between building blocks is required, so as to construct new materials more efficiently.

Secondly, many elemental and binary layered materials have shown that the thickness is essential for their property evolution when they are thinned down to a 2D limit. For ternary materials, such investigation is scarce and only performed in several vdW ternary materials including ZrNCl, FePS_3_, and MnBi_2_Te_4_. There are many ternary solids with a quasi-2D structure exhibiting unique electronic structures and properties, but strong interlayer interaction impedes the study of layer-dependent properties, such as infinite-layer nickelate RENiO_2_ (RE = rare earth metal) and Z2 topological kagome metal *A*V_3_Sb_5_ (A = alkaline metal) [139–141]. Therefore, the thickness-dependent property evolution of non-vdW ternary materials requires further investigation in both theoretical and experimental views. Except for homogeneous 2D materials, the exploration of heterostructures based on ternary layered materials is important in various fields including semiconductor devices, twistronics, and energy conversion. The heterostructures can be constructed with different geometric configurations (planar or vertical structures) and different material species and numbers of stacking layers. They can also be produced by vdW and non-vdW layers, which can be used to investigate the effect of strong interlayer interaction to modulate the properties of heterostructures.

Thirdly, controllable growth of 2D ternary layered materials is challenging. Ternary materials contain 3 elements and at least 3 atomic layers in a monolayer. During the growth process, 3 elements result in numerous combinations and generate diverse compounds with different stoichiometric ratios. How to choose a suitable precursor and design a reliable reaction path is essential to realize high-purity materials with a targeted stoichiometry. Moreover, multiple elements will lead to more defects and vacancies, especially when 2 elements of a ternary compound are close in atomic size. Amounts of antisite defects and grain boundaries will be formed in materials such as MnBi_2_Te_4_ and MA_2_Z_4_ [24,79,142]. Therefore, it is important to deeply understand the growth thermodynamics and kinetics of 2D ternary materials so as to precisely control atomic arrangement during the growth process, and novel solid-phase growth methods should be developed. Interestingly, from the existing synthetic routes of alloyed 2D binary materials, we can learn a lot in the control of atomic arrangement, which is beneficial in the growth of 2D structures of ternary layered materials, especially in the aspects of defect control and ordered construction of atomic layers. For example, in order to synthesize 2D 111-type compounds with 2 kinds of anions, layered intermediates can be formed with one layer substituted by atoms that are reactive, such as H atoms. Subsequent etching and replacement are preceded in such intermediates by volatile nonmetallic elements, so as to form ordered and well-defined 111-type ternary nanosheets.

In addition, many non-vdW ternary materials can be exfoliated by etching their chemically active layers to produce the 2D counterpart. It not only produces atomic-thickness nanosheets, but also creates novel 2D materials that do not exist in 3D bulk. However, it is difficult to achieve 2D nanosheets with a composition and structure consistent with bulk after exfoliation. For example, how to produce single-layer and few-layer MAX nanosheets rather than MXene needs new synthesis methods. Such nanosheets can help us to experimentally study the layer-dependent properties of non-vdW ternary materials. One way to achieve the goal is to control the removal of A content during the etching process. For example, electrochemical etching can synthesize AMX_2_ nanosheets with A layers preserved through controlling the etching voltage, but currently, only when A is Ag or Cu can it lead to exfoliation [39]. Thus, developing electrochemistry-assisted exfoliation for other non-vdW ternary materials will be necessary to produce 2D nanosheets with controlled A contents. Another solution synthetic strategy, mechanical force-assisted liquid exfoliation, including sonication- and ball milling-assisted exfoliation, should also be considered, since it can massively produce 2D materials with low cost and high quality [143–145]. Considering the key factor for achieving efficient exfoliation is the surface energy difference between the layer material and the solvent, it is essential to find a suitable solvent to match the surface energy of ternary layered materials. In addition, how to pretreat the bulk before sonication for high-efficiency exfoliation is another important question that requires consideration.

Moreover, defect chemistry will be much more complicated in ternary layered materials than in elemental and binary solids. The obtained 2D structures possess abundant defect types, including various types of vacancies, interstitial atoms, and grain boundaries and stacking faults. In addition, for 2D non-vdW ternary materials such as 112-type compounds and MXenes, large amounts of low-coordinated atoms and unsaturated dangling bonds exist at their surface and interface. Therefore, more effort should be put into the effect of defect on the intrinsic properties and practical performance of 2D ternary materials, especially in the fields of electronics and catalysis.

Finally, the exploration of potential applications deserves further investigation for 2D ternary layered materials. There are many 2D ternary compounds being predicted and uncovered with high performance and potential in semiconducting devices, optoelectronics, and spintronics, such as Bi_2_O_2_Se with high electron mobility and Fe_3_GeTe_2_ with robust ferromagnetism [17,146]. Future experiments should also be extended for the considerable development of thermal science and applications. Considering that many ternary layered materials contain mobile ionic layers and electron-conducting layers, especially ternary ionic conductors, their 2D materials and films show promise in pyroelectricity and thermal insulator applications. Furthermore, the elemental compositions of ternary layered materials are rich and cover many catalytic elements (Mn, Fe, Ni, Pd, Pt, etc.). Further studies are required to design and discover 2D ternary materials with high catalytic performance in water electrolysis, fuel cells, and small-molecule reduction reaction. We hope that this perspective can promote the exploration of these new members of the 2D family and help the precise design and synthesis with desired properties and applications.
